# Association between salivary s-IgA concentration and dental caries: an updated meta-analysis

**DOI:** 10.1042/BSR20203208

**Published:** 2020-12-22

**Authors:** Zeyu Wu, Yi Gong, Chen Wang, Jing Lin, Jin Zhao

**Affiliations:** 1 Department of Cariology and Endodontics, The First Affiliated Hospital of Xinjiang Medical University (The Affiliated Stomatology Hospital of Xinjiang Medical University), No. 137 South Liyushan Road, Urumqi 830054, People’s Republic of China; 2 Stomatology Disease Institute of Xinjiang Uyghur Autonomous Region, No.137 South Liyushan Road, Urumqi 830054, People’s Republic of China

**Keywords:** case-control study, Dental caries, Meta-analysis, s-IgA, Salivary

## Abstract

Objective: To determine the levels of s-IgA in saliva of caries patients and healthy controls, and to evaluate whether there is a correlation between it and caries by meta-analysis. Methods: The PubMed, MEDLINE, EMBASE, Web of Science, Cochrane Library, Scopus, Chinese National Knowledge Infrastructure, Wanfang Data, Chongqing VIP database for Chinese Technical Periodicals, and China BioMedical Literature Services System databases were searched initially in April 2020 and repeated in August 2020. Two independent evaluators screened the literature and extracted the data according to the inclusion and exclusion criteria. R 4.0.2 software was used for meta-analysis. *I*^2^ test was commonly reflected the heterogeneity. Subgroup analysis and meta-regression analysis explore the sources of heterogeneity. Sensitivity analysis, funnel diagram, Begg’s rank correlation, and Egger’s linear regression were used to determine the possibility of publication bias. Results: The study was reviewed according to the project guidelines for optimal reporting (PRISMA) based on meta-analysis. A total of 30 case–control studies were included, with a total sample size of 1545 patients, including 918 caries patients and 627 healthy controls. Salivary s-IgA levels in caries patients were significantly lower than those in healthy controls (SMD = −0.49, 95%CI: [−0.94; −0.03], *P*=0.03). In addition, the results of subgroup analysis showed that the significant decrease of salivary s-IgA level was correlated with children patients, mixed dentition and Asian people (children: SMD = −0.45, 95%CI: [−0.89; −0.01], *P*=0.04; mixed dentition: SMD = −0.61, 95%CI: [−1.24; 0.03], *P*=0.06; Asian: SMD = −0.62, 95%CI: [−1.17; −0.08], *P*=0.02). The funnel diagram included in the study was symmetrically distributed, and the sensitivity analysis confirmed the robustness of the results. Conclusion: Salivary s-IgA levels in caries patients were significantly lower than in healthy controls. It has also been demonstrated that salivary s-IgA may be used as an alternative measure to identify subjects at risk of caries susceptibility, suggesting that salivary s-IgA may be a protective factor for dental caries.

## Introduction

Caries remains a serious public health problem in most parts of the world, about 10 percent of the world’s people suffering from this disease [1]. It is a kind of disease that the bacteria in the dental plaque ferment the sugar in the food to produce the acid, cause the tooth hard tissue demineralization dissolves and produce the chronic progressive destruction [2]. Saliva contains a variety of components, including electrolytes, enzymes, and antimicrobial peptides such as immunoglobulin A, lactoferrin, and lysozyme [3]. Saliva is involved in a variety of physiological functions, including lubricating the mouth, wetting food and swallowing, protecting the oral mucosa from dryness, participating in immune defense, and playing a key role in regulating the ecological balance of oral flora [4]. In particular, salivary secretory immunoglobulin A (s-IgA) not only mediates the humoral immune response to regulate caries activity but also interferes with the formation of caries causing microbial adhesion to the tooth surface and biofilm [5]. Studies in recent years have shown that salivary s-IgA plays an important role in local immunity by binding to caries causing microbial surface molecules such as adhesion to prevent microbial adhesion to the surface of tooth hard tissue [6]. It can reduce the hydrophobicity of the surface of bacteria and directly destroy *Streptococcus mutans* toxin and glucosyltransferase (GTF), making them inactivated [7]. Directly combine with the cariogenic microorganism to form an immune complex, which is more beneficial to remove [7]. Salivary s-IgA acts together with complement lysozyme to dissolve bacteria [7]. Regulate the phagocytic function of mucosa polynuclear leukocytes and phagocytes.

The locally specific immune response mediated by s-IgA is species-specific and can induce cross-reactive immunity to enhance the original local immunity [8]. Salivary s-IgA, combined with specific epitopes of cariogenic bacteria, resulted in a locally specific immune response. In recent years, several studies have assessed the relationship between aggregate, nonspecific, and specific levels of s-IgA in saliva and caries, but the results from different studies vary widely. One theory is that salivary s-IgA inhibits the process of bacterial adhesion to the tooth surface, neutralizes certain enzymes and bacterial toxins of cariogenic bacteria, and synergizes with other salivary proteins such as lactoferrin or lysozyme, which may have caries-preventing effects [7,9,10]. In contrast, another theory suggests that differences in salivary s-IgA levels have nothing to do with caries susceptibility. People with higher salivary s-IgA levels have more dental caries than those with lower levels, suggesting a protein concentration-effect [10,11].

A previous meta-analysis found that the salivary s-IgA levels of patients with dental caries were higher than that of healthy controls, but the results of the study were highly heterogeneous and did not explore the source of the heterogeneity [12]. As time goes by, the results of most studies in recent years have shown the opposite conclusions of the previous meta-analysis. To further explore the relationship between salivary s-IgA levels and caries, we conducted the present study. We excluded noncompliant literature and reported an updated meta-analysis of all studies of salivary s-IgA levels, and performed subgroup analysis, sensitivity, and meta-regression analysis to explore heterogeneity sources. Publication bias was identified by using the funnel graph method, Begg’s rank correlation, and Egger’s linear regression. Therefore, by collecting relevant literature on salivary s-IgA levels and caries before June 2020, meta-analysis was conducted in this paper to compare the differences in salivary s-IgA levels between caries-free groups and dental caries patients. Provide help for early clinical intervention and prevention of dental caries patients, and provide a reference basis for a screening of dental caries susceptible population.

## Materials and methods

### Protocol and registration

The Preferred Reporting Items for Systematic Review and Meta-Analysis (PRISMA) checklist was used as a guide for conducting and reporting the present study (Supplementary Table S1) [13]. This updated meta-analysis was registered on the International Prospective Register of Systematic Reviews (PROSPERO) database (registry number: CRD42018112317; http://www.crd.york.ac.uk/PROSPERO/display_record.php?ID=CRD42018112317).

### Selection criteria

#### Inclusion criteria

(1) A case–control study of salivary secretory immunoglobulin A measurements in caries and control cases published inside and outside China as of August 2020. (2) Without language limitation. (3) Clinical investigation of both case (caries group) and control (caries-free group). (4) Salivary levels of s-IgA were measured in both the case and control groups. (5) Caries was diagnosed by the World Health Organization (WHO) according to DMFT/dmft or DMFS/dmfs index as the standard.

#### Exclusion criteria

(1) There are no specific data report or the data are too small, which is not conducive to the analysis of literature; 2) Repeat study; 3) Only case group (caries group), no control literature; 4) Animal experiments and *in vitro* studies; 5) A history of congenital malformations and systemic diseases; 6) Literature types included case report, review, systematic evaluation, etc.

### Search strategy

Through domestic databases: Chinese National Knowledge Infrastructure (CNKI), Wanfang Data, Chongqing VIP database for Chinese Technical Periodicals (VIP), and China BioMedical Literature Services System (SinoMed); Foreign databases: PubMed, MEDLINE, Embase, Scopus, Web of Science, Cochrane Library. The search process is completed by two evaluators independently under the guidance of professionals. Subjects and free word searches were performed using the following keywords: ‘Dental Caries’, ‘Immunoglobulin A, Secretory’, ‘Immunoglobulin A’, ‘Saliva’. According to the references found after the literature take the manual search, to prevent the omission of important literature. The retrieval strategy is shown in the Table 1.

**Table 1:** Search strategy

No.	Query	Results
#12	Search #3 AND #8 AND #11	360
#11	Search #9 OR #10	61724
#10	((Saliva [Title/Abstract]) OR (Salivas [Title/Abstract])) OR (spittle [Title/Abstract])	45519
#9	Search “Saliva”[Mesh]	41626
#8	Search #4 OR #5 OR #6 OR #7	68162
#7	(((Immunoglobulin A [Title/Abstract]) OR (IgA [Title/Abstract])) OR (IgA1 [Title/Abstract])) OR (IgA2 [Title/Abstract])	55797
#6	Search “Immunoglobulin A” [Mesh]	37402
#5	((((((((Immunoglobulin A, Secretory [Title/Abstract]) OR (Secretory IgA [Title/Abstract])) OR (IgA, Exocrine [Title/Abstract])) OR (Exocrine IgA [Title/Abstract])) OR (SIgA [Title/Abstract])) OR (IgA, Secretory [Title/Abstract])) OR (Secretory Immunoglobulin A [Title/Abstract])) OR (Colostral IgA [Title/Abstract])) OR (IgA, Colostral [Title/Abstract])	6131
#4	Search “Immunoglobulin A, Secretory” [Mesh]	5611
#3	Search #1 OR #2	62212
#2	(((((((((((((((((Dental Caries [Title/Abstract]) OR (Dental Decay [Title/Abstract])) OR (“Caries, Dental” [Title/Abstract])) OR (“Decay, Dental” [Title/Abstract])) OR (Carious Dentin [Title/Abstract])) OR (Carious Dentins [Title/Abstract])) OR (“Dentin, Carious” [Title/Abstract])) OR (“Dentins, Carious” [Title/Abstract])) OR (Dental White Spot [Title/Abstract])) OR (“White Spots, Dental” [Title/Abstract])) OR (White Spots [Title/Abstract])) OR (“Spot, White” [Title/Abstract])) OR (“Spots, White” [Title/Abstract])) OR (White Spot [Title/Abstract])) OR (Dental White Spots [Title/Abstract])) OR (“White Spot, Dental” [Title/Abstract])) OR (caries [Title/Abstract])) OR (tooth caries [Title/Abstract])	47252
#1	Search “Dental Caries” [Mesh]	46062

Source: PubMed; Searched on August 8, 2020

### Literature screening and data extraction

Repeated references were deleted by using the EndNote X9 software. Literature and data will be selected and extracted by two reviewers independently. If there is any disagreement, it will be decided by discussion or by the third evaluator. We reviewed each literature using the exclusion and inclusion criteria. If the same author uses the same data again in another study, only one study will be included. In the literature screening, the title of the article was read first, and the abstract was read further after excluding irrelevant literature to determine whether to include or not. Then read the full text in detail, culling irrelevant articles. The data were extracted into pre-designed forms as follows: first author’s name, publication date, country, age, sample size, caries index (dmft and/or DMFT), s-IgA content, saliva extraction time, saliva type, s-IgA measurement type, and statistical methods and results. After data extraction, the two data were checked, inconsistencies were extracted again, and data analysis was conducted after confirmation. If the included studies provided only the median and extreme values of salivary s-IgA levels, they could be converted to mean and standard deviation according to the methods provided by Wan et al [14]. If the original article does not provide any important data for the above characteristics, we are attempting to send an email to the author to obtain available information.

### Quality assessment

The included studies were assessed using the case–control study quality assessment criteria recommended by the Newcastle–Ottawa scale (NOS) criteria [15]. At present, this was the only accepted quality assessment tool in the case–control study, see the supplementary document. Supplementary Table S4 lists the NOS case–control study quality assessment table. The scale allows for the evaluation of case–control, cohort, and observational studies. The scale provides a standardized method for quality evaluation, with a total of 8 items, including the selection of research objects, comparability of study groups, and exposure or outcome ascertainment. The stars are used to answer the question, and with a maximum score of 9 for each study. The quality evaluation was conducted by two researchers independently at the same time, and a third party would decide the differences after discussion. The quality of the literature was determined by stars number: 0–3 stars were of low quality, 4–6 stars were of medium quality, and 7–9 stars were of high quality [16].

### Statistical analysis

Data were analyzed by a third researcher using R 4.0.2 software. Mean concentration, standard deviation, and sample size of salivary s-IgA were used for statistical analysis. Due to the different units of salivary s-IgA concentration in each study, all the measured results were converted to μg/ml. Values from the baseline were used in studies evaluating baseline and follow-up. For the evaluation of low, medium, and high caries patients, we used the combined subgroup method to obtain the salivary s-IgA value. Subgroup analyses were also performed, dividing the study into region differences (Asia, Europe, North America, South America, Africa), age differences (adults, ≥18; children, <18), dentition type (permanent dentition, mixed dentition, deciduous dentition), salivary s-IgA detection methods (enzyme-linked immunosorbent assay (ELISA), Immunoturbidimetry, radioimmunoassay (RIA), noncompetitive biotin-avidin enzyme-linked immunosorbent assay (NABA)). Continuous variable data were used as the effective index, and the results were expressed as mean difference (MD) or standardized mean difference (SMD) effect quantity and 95% confidence interval (CI). Heterogeneity of the included results was analyzed by the χ2 test (test level: *α* = 0.01), and the quantitative judgment of heterogeneity was made by combining *I*^2^ [17]. When *I*^2^>50%, indicating that there was significant heterogeneity among the studies included in the meta-analysis, and the random effect model was used for statistical analysis of the summary results of the present study. Sensitivity analysis was used to evaluate the reliability of the overall effect at all levels and to find out the factors affecting the inter-study heterogeneity as far as possible. To determine the source of heterogeneity, stratified subgroup analysis, and random effect meta-regression were performed by age, dentistry, region, and salivary s-IgA assays to assess potential confounders in the present study. Begg’s [18] and Egger’s [19] tests and funnel charts were used to assess the potential publication bias in the included studies.

## Results

### Inclusion literature screening

EndNote X9 literature management software was used to merge the retrieval results, and 1320 duplicate results were removed, leaving 920 for study. After reading the title and abstract, 222 studies were excluded for reasons including reviews, *in vitro* studies or animal experiments, indicators that did not include salivary s-IgA levels, duplicate reports, incorrect or incomplete data, and unreasonable study design.

**Figure 1:**
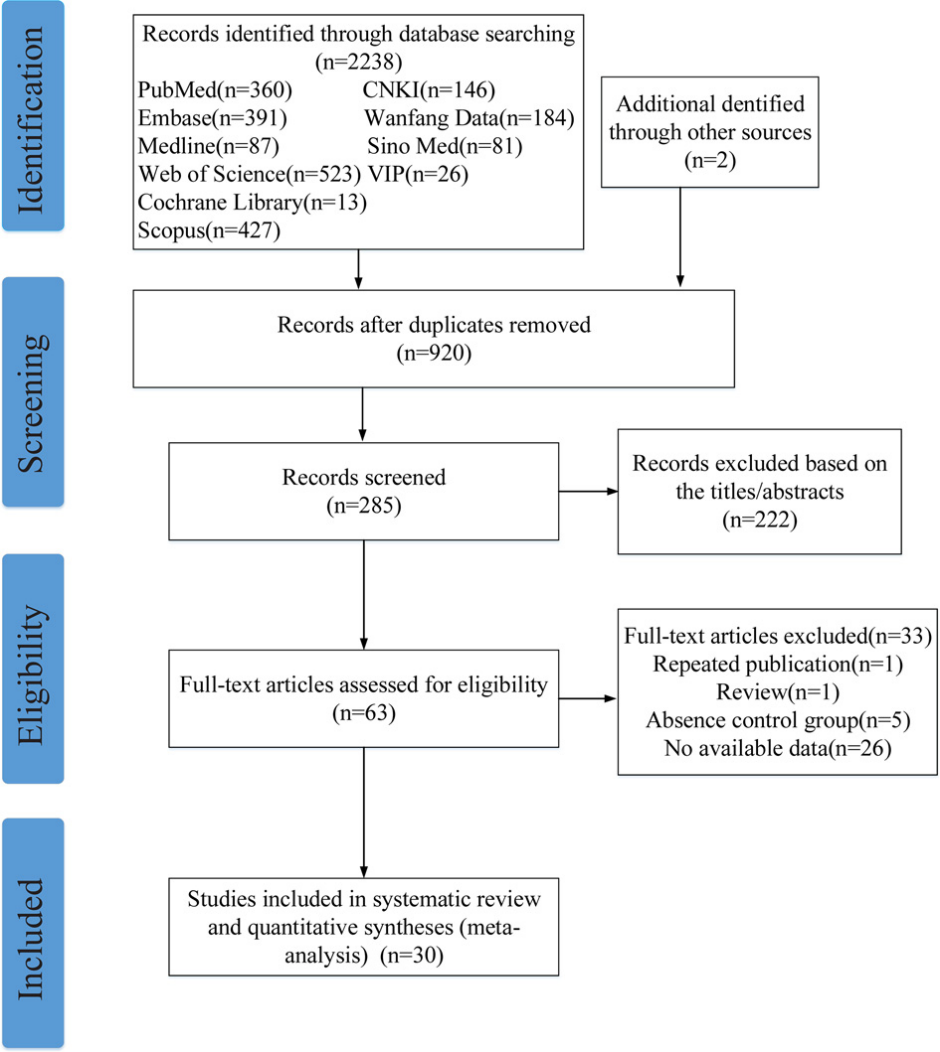
Flow chart showing the study selection

**Figure 2:**
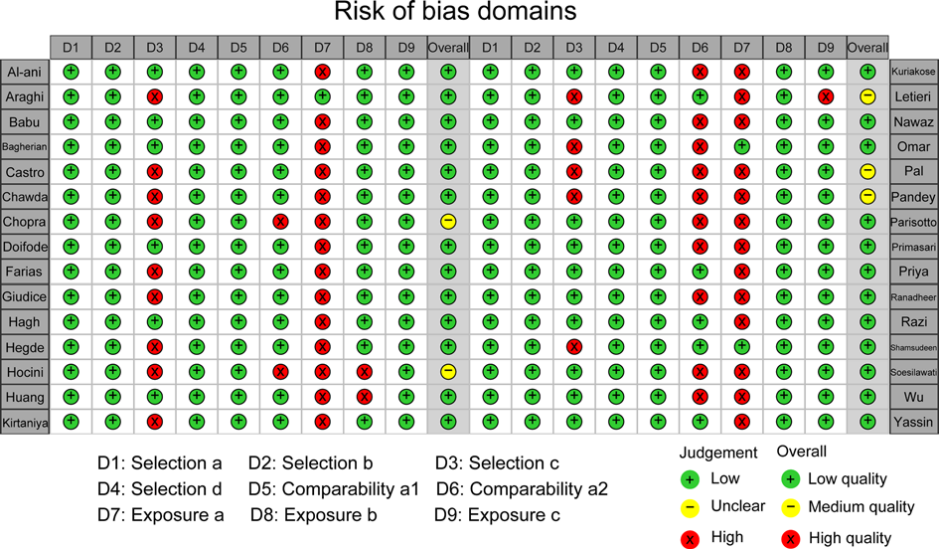
Risk of bias assessment for included studies Reviewers’ judgment of the risk of bias for each item for each of the 30 studies included in the meta-analysis.

About 63 studies were screened by two reviewers independently and blind, and 30 studies were finally included [5]. The screening process is shown in Figure 1. The total sample size of the included study was 1545 cases, 918 cases in the caries group, and 627 cases in the control group. Information on the included studies is shown in Table 2. The included studies were published between 1993 and 2020. All studies were case–control and reported the relationship between salivary s-IgA levels and dental caries in different age groups and different dentition period. Table 3 shows the nature of saliva collected in each study, collection time, the method for detecting s-IgA, and the basic characteristics of statistical analysis. Figure 2 shows the evaluation results of 30 studies in the NOS case-control study quality evaluation form.

**Table 2:** Characteristics of included studies

Author and year	Country	Subject							
		Caries-active				Caries-free			
		Age (year)	Sample size	Caries index (*n*)	s-IgA level (μg/ml)	Age (year)	Sample size	Caries index	s-IgA level (μg/ml)
Al-ani 2020 [20]	U.S.A.	18–70 (38.36 ± 13.20)	All: 28 Male: 13 Female: 15	High caries risk (28)	All: 0.207 ± 0.133 Male: 0.2414 ± 0.14817 Female: 0.17699 ± 0.11394	18–70 (38.84 ± 12.16)	All: 32 Male: 14 Female: 18	Low caries risk	All: 0.238 ± 0.139 Male: 0.26016 ± 0.17057 Female: 0.21996 ± 0.11159
Araghi 2018 [21]	IRN	20–40	All: 30 Low caries: 20 High caries: 10	DMFT <5 (20) DMFT ≥5 (10)	All: 0.1472 ± 0.071 Low caries: 0.1328 ± 0.0509 High caries: 0.1760 ± 0.0968	20–40	10	DMFT = 0	0.0939 ± 0.0239
Babu 2017 [22]	IND	8–12 (10.00 ± 1.33)	20	DMFT = 3.8 ± 1.00	158.55 ± 30.24	8–12 (8.9 ± 0.71)	20	DMFT = 0	183.80 ± 19.37
Bagherian 2013 [23]	IRN	3–5.8 (5.08 ± 0.73)	45	dmft = 9.3 ± 3.6	1961.4 ± 1000.7	3–5.83 (4.92 ± 1.02)	45	dmft = 0	1484.5 ± 811.6
Castro 2016 [24]	CHL	25 ± 3	20	DMFT = 3.9 ± 0.7	26.80 ± 2.5	24 ± 2	20	DMFT = 0	50.65 ± 7.5
Chawda 2011 [25]	IND	4–8	All: 20 Low caries: 10 High caries: 10	DMFT+dmft = 1-5 (10) DMFT+dmft = 6-10 (10)	All: 176.45 ± 40.692 Low caries: 186.6 ± 48.4 High caries: 166.3 ± 30.4	4–8	10	DMFT+dmft = 0	243.6 ± 48.7
Chopra 2011 [26]	IND	24–45	88	DMFT > 1	774 ± 473	24–45	14	DMFT = 0	727 ± 409
Doifode 2011 [27]	IND	8–10 (8.73 ± 0.46)	15	dfs = 19.00 ± 8.52 DMFS = 1.60 ± 2.03	89.8 ± 15.6	8–10 (9.13 ± 0.35)	15	dfms = 0 DMFT = 0	107.4 ± 15.2
Farias 2003 [28]	BRA	1–3.9 (3.14 ± 0.75)	20	dmfs = 16.4 ± 8.9	50.4 ± 45.0	1–3.9 (3.29 ± 0.59)	20	dmft = 0	32.5 ± 21.0
Giudice 2019 [29]	ITA	4–16	39	DMFT+dmft = 0.25 ± 0.24	218.0 ± 129.0	4–16	20	DMFT+dmft = 0	167.0 ± 45.0
Hagh 2013 [30]	IRN	19–4 (26.8 ± 5.61)	15	DMFT = 5.26 ± 3.04	6.02 ± 0.76	19–4 (28.5 ± 7.07)	25	DMFS = 0	12.32 ± 1.99
Hegde 2013 [5]	IND	20–30	All: 60 Low caries: 20 High caries: 40	DMFT = 1–5 (20) DMFT = 6–10 (20) DMFT ≥ 10 (20)	All: 1905.10 ± 1016.57 High caries: 1384.6 ± 609.14 Low caries: 2946.10 ± 858.10	20–30	20	DMFT = 0	3155.1 ± 489.3
Hocini 1993 [31]	FRA	20-63 (34.3 ± 10.7)	All: 21 Male: 11 Female: 10	DMFT > 10	All: 34.2 ± 20.9 Male: 35.53 ± 18.3 Female: 32.7 ± 24.37	22–64 (38.5 ± 11.8)	All: 22 Male: 13 Female: 9	DMFT = 0	All: 31.4 ± 36.1 Male: 36.15 ± 45.61 Female: 24.58 ± 14.33
Huang 2006 [32]	CHN	4–6	45	dmft ≥ (4–6)	1195.7 ± 538.2	4–6	45	dmft = 0	2272.0 ± 673.1
Kirtaniya 2009 [33]	IND	6–14	All: 35 Low caries: 20 High caries: 15	DMFT+deft = 1.7 ± 0.48 (10) DMFT+deft = 3.6 ± 0.52 (10) DMFT+deft = 8.8 ± 3.49 (15)	All: 0.3819 ± 0.1224 Low caries: 0.4095 ± 0.1015 High caries: 0.345 ± 0.141	6–14	11	DMFT+deft = 0	0.49 ± 0.142
Kuriakose 2013 [34]	IND	3–5	17	dmft > 5	99.6 ± 28.3	3–5	17	dmft = 0	151.5 ± 22.2
Letieri 2019 [35]	BRA	2–5 (3.0 ± 1.0)	23	dmfs = 10.2	32.94 ± 32.16	2–5 (3.7 ± 1.2)	23	dmfs = 0	25.40 ± 15.44
Nawaz 2019 [36]	PAK	33 ± 12.58	All: 28 Low caries: 29 High caries: 29	DMFT = 0–5 (29) DMFT > 5 (29)	All: 32.165 ± 8.0561 Low caries: 34.64 ± 6.37 High caries: 29.69 ± 8.88	30 ± 7.3	29	DMFT = 0	7.67 ± 8.23
Omar 2012 [37]	EGY	4–6	35	dmft = 1–3 (11); dmft = 4–6 (13);dmft > 6 (11)	All: 0.7514 ± 0.3946	4–6	10	dmft = 0	0.81 ± 0.38
Pal 2013 [38]	IND	9.5 ± 2.45	All: 30 Low caries: 15 High caries: 15	DMFT = 6.600 ± 2.098 DMFT = 2.200 ± 0.941	All: 167.635 ± 31.8001 Low caries: 189.47 ± 25.99 High caries: 145.80 ± 19.94	10.00 ± 2.59	15	DMFT = 0	215.4 ± 26.71
Pandey 2018 [39]	IND	5–14 (9.36 ± 2.37)	All: 40 Low caries: 20 High caries: 20	DMFT = 2.32 ± 0.86 (20) DMFT = 6.74 ± 2.16 (20)	All: 164.315 ± 32.0691 Low caries: 186.10 ± 24.70 High caries: 142.53 ± 22.4	5–14 (10.2 ± 2.35)	20	DMFT = 0	214.8 ± 27.56
Parisotto 2011 [40]	U.S.A.	3–4	17	dmfs ≥ 3 (dmft = 3.10 ± 2.6)	181.97 ± 34.18	3–4	23	dmfs = 0	132.22 ± 19.03
Primasari 2019 [41]	IDN	1.65 ± 0.44	All: 34 Low caries: 26 High caries: 8	deft = 1–3 (14); deft = 4–5 (18); deft = 6–8 (8)	All: 0.602 ± 0.424 Low caries: 0.6287 ± 0.4344 High caries: 0.514 ± 0.405	1.35 ± 0.39	34	dmft = 0	0.702 ± 0.421
Priya 2013 [42]	IND	7–12	15	DMFT/dmft ≥ 3	130.7 ± 15.5	7–12	15	DMFT/dmft = 0	119.0 ± 15.8
Ranadheer 2011 [43]	IND	8–12	20	DMFT/dmft ≥ 3	117.6 ± 18.5	8–12	20	DMFT/dmft = 0	75.85 ± 24.8
Razi 2020 [44]	IND	12–15	All: 20 Male: 10 Female: 10	DMFT ≥ 10	All: 85.0 ± 14.3 Male: 91.8 ± 8.3 Female: 79.1 ± 16.3	12–15	All: 20 Male: 10 Female:10	DMFT = 0	All: 106.3 ± 28.5 Male: 118.2 ± 34.3 Female: 94.3 ± 15.3
Shamsudeen 2008 [45]	IND	3–6	10	dmft ≥ 5	2211 ± 778.68	3–6	10	dmft = 0	2300.0 ± 432.0
Soesilawati 2019 [46]	IDN	6–9	All: 48 Low caries: 40 High caries: 8	dmft = 1 (15); dmft = 2 (3); dmft = 3 (3); dmft = 4 (19); dmft = 5 (8)	All: 295.63 ± 183.57 Low caries: 320.30 ± 187.75 High caries: 172.25 ± 94.80	6–9	12	dmft = 0	545.83 ± 90.30
Wu 2002 [47]	CHN	4–5	20	dmft ≥ 5	52.44 ± 13.23	4–5	20	dmft = 0	75.73 ± 22.15
Yassin 2016 [48]	IRQ	7–10	All: 30 Male: 11 Female: 19	DMFT/dmft ≥ 5	All: 1091.2 ± 287.8 Male: 1037.7 ± 242.0 Female: 1122.2 ± 313.3	7–10	All: 30 Male: 12 Female: 18	DMFT/dmft = 0	All: 1285.8 ± 281.0 Male: 1305.2 ± 291.7 Female: 1272.9 ± 281.4

DMFT/dmft index: the sum of decayed missing and filled permanent/deciduous teeth; DMFS/dmfs index: the sum of decayed missing and filled permanent/deciduous surfaces; Low caries: DMFT/dmft = 1–5; High caries: DMFT/dmft > 5.

**Table 3:** Characteristics of included studies and quality score

First Author and Year	Saliva collection	Time	Amount of saliva	Detection method	Statistic test and *P*-value	Quality score
Al-ani 2020 [20]	Unstimulated saliva	Missing data	5–10 ml	ELISA	Mann–Whitney and Kruskal–Wallis tests, *P*=0.388	8
Araghi 2018 [21]	Unstimulated saliva	10:00–12:00 (a.m.)	Missing data	Immunoturbidimetry	ANOVA test, *P*=0.046	8
Babu 2017 [22]	Unstimulated saliva	10:00–12:00 (a.m.)	2 ml	ELISA	T test, *P*=0.0399	8
Bagherian 2013 [23]	Unstimulated saliva	10:00–11:00 (a.m.)	Missing data	ELISA	Pearson test, *P*=0.015	8
Castro 2016 [24]	Unstimulated saliva	09:00–11:00 (a.m.)	15 ml	ELISA	Student’s *t* test, *P*=0.001	7
Chawda 2011 [25]	Unstimulated saliva	10:00–12:00 (a.m.)	0.5 ml	Immunoturbidimetry	ANOVA test, *P*=0.001	7
Chopra 2011 [26]	Unstimulated saliva	09:00–11:00 (a.m.)	Missing data	ELISA	ANOVA test, *P*>0.05	6
Doifode 2011 [27]	Unstimulated saliva	Missing data	Missing data	Immunoturbidimetry	*T* test, *P*=0.012	8
Farias 2003 [28]	Unstimulated saliva	08:00–11:00 (a.m.)	1.5 ml	Immunoturbidimetry	*U* Mann–Whitney, *P*<0.05	7
Giudice 2019 [29]	Unstimulated saliva	10:00–11:00 (a.m.)	Missing data	ELISA	Mann–Whitney, *P*=0.175	7
Hagh 2013 [30]	Unstimulated saliva	09:00–12:00 (a.m.)	Missing data	ELISA	Mann–Whitney and Kruskal–Wallis tests, *P*=0.009	8
Hegde 2013 [5]	Unstimulated saliva	10:00–11:00 (a.m.)	Missing data	Immunoturbidimetry	One-way ANOVA test, *P*<0.001	7
Hocini 1993 [31]	Unstimulated saliva	Missing data	Missing data	ELISA	Mann–Whitney *U* test, *P*>0.05	5
Huang 2006 [32]	Unstimulated saliva	10:00–11:00 (a.m.)	2 ml	RIA	Group *t* test, *P<*0.05	7
Kirtaniya 2009 [33]	Unstimulated saliva	09:00–12:00 (a.m.)	Missing data	ELISA	*P*<0.01	7
Kuriakose 2013 [34]	Unstimulated saliva	08:00–09:00 (a.m.)	5 ml	ELISA	Unpaired *t* test, *P*=0.001	7
Letieri 2019 [35]	Unstimulated saliva	08:00–10:00 (a.m.)	1 ml	ELISA	Mann–Whitney *U* test, *P*<0.03	6
Nawaz 2019 [36]	Unstimulated saliva	Missing data	3 ml	ELISA	ANOVA test, *P*<0.001	7
Omar 2012 [37]	Unstimulated saliva	09:00–11:00 (a.m.)	3 ml	ELISA	Low and high caries group, Pearson test, *P*<0.01	7
Pal 2013 [38]	Unstimulated saliva	10:00–11:00 (a.m.)	4 ml	ELISA	ANOVA test, *P*<0.001	6
Pandey 2018 [39]	Unstimulated saliva	10:00–11:00 (a.m.)	5 ml	ELISA	Chi-square test, *P*=0.001	6
Parisotto 2011 [40]	Unstimulated saliva	Missing data	250 μl	ELISA	Wilcoxon signed-rank test, *P*=0.0118	7
Primasari 2019 [41]	Unstimulated saliva	09:00–11:00 (a.m.)	2 ml	ELISA	Kruskal–Wallis ANOVA test, *P*=0.227	7
Priya 2013 [42]	Unstimulated saliva	Missing data	2–3 ml	ELISA	Independent sample *t* test, *P*=0.05	8
Ranadheer 2011 [43]	Unstimulated saliva	Missing data	Missing data	ELISA	Schefft test, *P*=0.05	7
Razi 2020 [44]	Unstimulated saliva	Missing data	5 ml	ELISA	Independent *t* test, *P*=0.015	8
Shamsudeen 2008 [45]	Unstimulated saliva	10:00–11:00 (a.m.)	Missing data	Immunoturbidimetry	Student’s *t* test, *P*>0.05	8
Soesilawati 2019 [46]	Unstimulated saliva	10:00–12:00 (a.m.)	Missing data	ELISA	Mann–Whitney *U* test, *P*<0.001	7
Wu 2002 [47]	Unstimulated saliva	09:00–10:00 (a.m.)	2 ml	NABA	Group *t* test, *P<*0.05	7
Yassin 2016 [48]	Unstimulated saliva	Missing data	Missing data	Immunoturbidimetry	Group *t* test, *P*=0.01	8

Abbreviations: ELISA, enzyme-linked immunosorbent assay; NABA, noncompetitive avidin-biotin immunoenzymatic assay; RIA, radioimmunoassay.

### The correlation between salivary s-IgA level and caries

**Figure 3:**
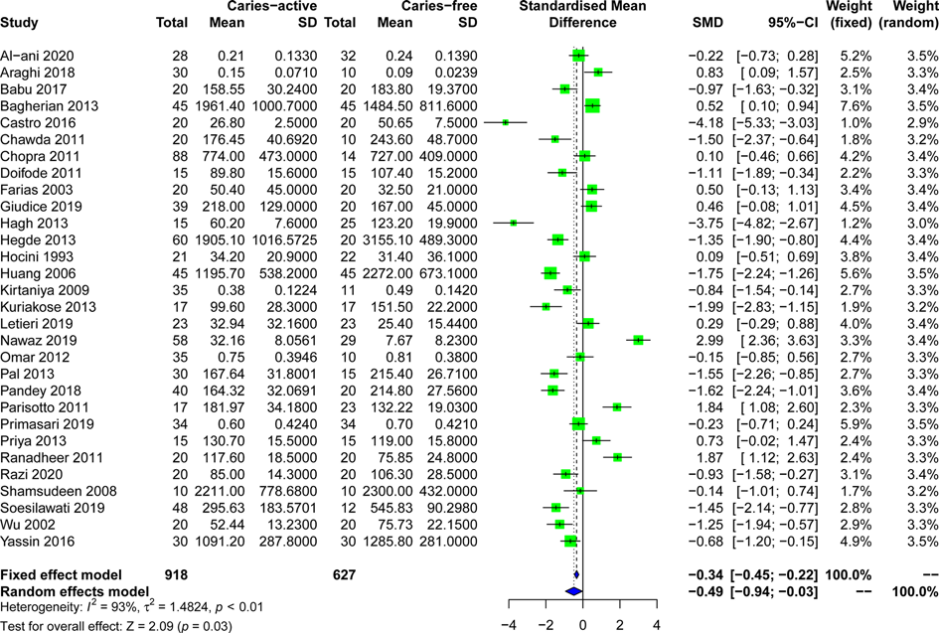
Forest plots of salivary s-IgA levels in patients with dental caries and control groups

A total of 30 studies reported salivary s-IgA levels in caries patients. The overall results of the correlation between salivary s-IgA levels and caries were shown in Figure 3. Salivary s-IgA levels in caries patients (mean ± SD) ranged from 2211.00 ± 778.68 [45] to 0.1472 ± 0.0710 μg/ml [21], while those in healthy controls ranged from 3155.10 ± 489.30 [5] to 0.0939 ± 0.0239 μg/ml [21]. Due to the heterogeneity of *I*^2^>50% in the study, the random effect model (*P*<0.001) was adopted for data consolidation analysis. The negative correlation between salivary s-IgA levels and caries was determined in this meta-analysis (SMD = −0.49, 95%CI: [−0.94; −0.03], *P*=0.03; Figure 3).

### Subgroup analysis

To explore the source of heterogeneity of differences in salivary s-IgA levels, we based on region differences, age differences, dentition type, and salivary s-IgA detection methods were sub-group analysis (Table 4). First, the subgroup analysis based on region (Table 4 and Supplementary Figure S1), due to the obvious heterogeneity (*I*^2^>50%), the random-effects model was adopted, showing that the salivary s-IgA levels of the Asian caries group were significantly lower than that of the healthy control group (SMD = −0.62, 95%CI: [−1.17; −0.08], *P*=0.02), and people in other regions did not show significant differences. Based on the age subgroup analysis (Table 4 and Supplementary Figure S2), due to the obvious heterogeneity, the random-effects model was adopted, showing that the salivary s-IgA levels of the children’s caries group were significantly lower than that of the healthy control group (SMD = −0.45, 95%CI: [−0.89; −0.01], *P*=0.04), and adults did not show significant differences. However, the number of studies on adults was limited, so further research is needed to confirm this finding. Based on the subgroup analysis of dentition (Table 4 and Supplementary Figure S3), due to the obvious heterogeneity, the random-effects model was used to show that the salivary s-IgA levels of caries patients with mixed dentition were lower than that of the healthy control group (SMD = −0.61, 95%CI: [−1.24; 0.03], *P*=0.06), but the difference was not significant. There was no difference in salivary s-IgA levels of caries patients with permanent dentition and deciduous dentition compared with healthy controls. The subgroups were ELISA, immunoturbidimetry, RIA, and NABA, but showed that salivary s-IgA levels have no significant difference between dental caries patients and healthy controls (Table 4 and Supplementary Figure S4). Subgroup analysis showed that age and region might be the source of salivary s-IgA levels heterogeneity.

**Table 4:** Subgroup analysis of salivary s-IgA levels in dental caries patients

Subgroups	*N*	SMD	SMD (95%CI)	*Z*	*P*	Model	Heterogeneity	
							*I*^2^	*P*
**Salivary s-IgA**								
**Area**								
North America	100	0.79	−1.24; 2.81	0.77	0.44	Random	95.1%	<0.001
Asia	1172	−0.62	−1.17; −0.08	2.26	0.02	Random	93.9%	<0.001
South America	126	−1.06	−3.27; 1.14	0.95	0.34	Random	96.4%	<0.001
Europe	102	0.30	−0.11; 0.70	1.46	0.14	Fixed	0%	0.363
Africa	45	−0.15	−0.85; 0.56	0.42	0.68	Random	NA	NA
**Combined**	1545	−0.49	−0.94; −0.03	2.11	0.03	Random	93.6%	<0.001
**Age**								
≥18	492	−0.64	−1.92; 0.64	0.99	0.32	Random	96.7%	<0.001
<18	1053	−0.45	−0.89; −0.01	2.00	0.04	Random	90.4%	<0.001
**Combined**	1545	−0.49	−0.94; −0.03	2.11	0.03	Random	93.6%	<0.001
**Dentition periods**								
Deciduous dentition	513	−0.23	−0.91; 0.44	0.68	0.50	Random	92.2%	<0.001
Mixed dentition	500	−0.61	−1.24; 0.03	1.86	0.06	Random	90.3%	<0.001
Permanent dentition	532	−0.66	−1.81; 0.48	1.15	0.25	Random	96.4%	<0.001
**Combined**	1545	−0.49	-0.94; -0.03	2.11	0.03	Random	93.6%	<0.001
**Detection method**								
ELISA	1135	−0.39	−0.97; 0.19	1.33	0.18	Random	94.4%	<0.001
Immunoturbidimetry	300	−0.49	−1.16; 0.17	1.45	0.15	Random	85.5%	<0.001
RIA	90	−1.75	−2.24; −1.26	7.08	<0.001	Random	NA	NA
NABA	40	−1.25	−1.94; −0.57	3.66	<0.001	Random	NA	NA
Combined	1545	−0.49	−0.94; −0.03	2.11	0.03	Random	93.6%	<0.001

CI, confidence interval; ELISA, enzyme-linked immunosorbent assay; *N*, number of studies; NA, not available; NABA, noncompetitive avidin-biotin immuno enzymatic assay; RIA, radioimmunoassay; SMD, standardized mean difference.

### Salivary s-IgA level and the different gender of dental caries patients

**Figure 4:**
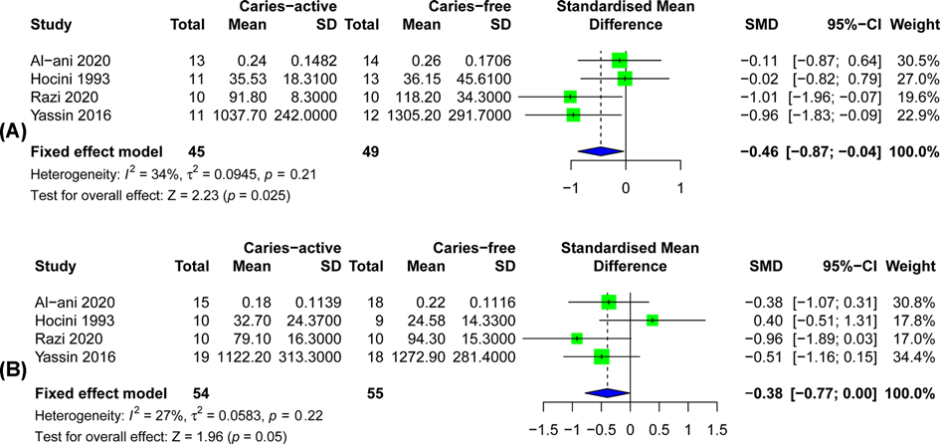
Forest plots of salivary s-IgA levels in different gender patients with dental caries and control groups (**A**) Forest plot of the relationship between salivary s-IgA levels and male group. (**B**) Forest plot of the relationship between salivary s-IgA levels and female group.

The results of the correlation between salivary s-IgA levels and gender were shown in Figure 4. Because of the limited number of studies on gender differences (only four studies were included) [20,31,44,48], and the heterogeneity in the studies was less than 50% (*I*^2^<50%), the fixed effects model was used (*P*>0.05). When comparing the salivary s-IgA levels of male caries patients and healthy controls (Figure 4A), we found that the salivary s-IgA levels of male caries patients were significantly lower than that of healthy controls (SMD = −0.46, 95%CI: [−0.87; −0.04], *P*=0.025). Similar results were found in women (Figure 4A). The salivary s-IgA levels of the caries group were lower than that of the healthy control group, but the difference was not statistically significant (SMD = −0.38, 95%CI: [−0.77; 0.00], *P*=0.05).

### The relationship between the saliva s-IgA level and the severity of dental caries in patients with dental caries

**Figure 5:**
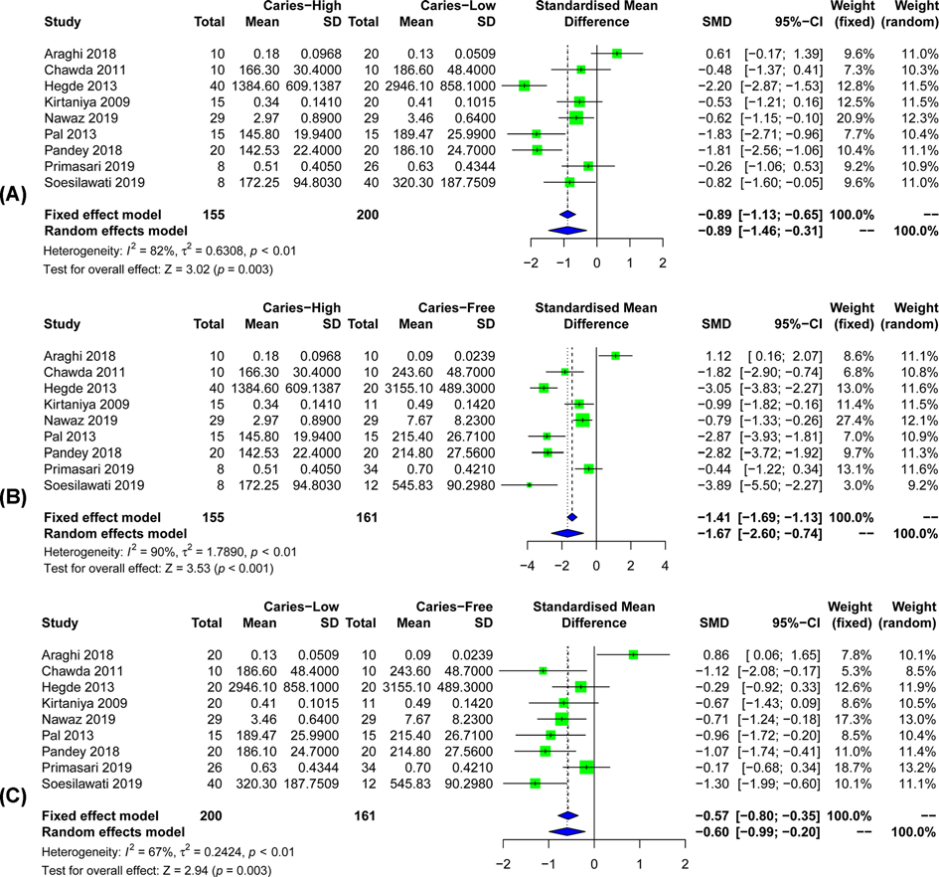
Forest plots of salivary s-IgA levels in different caries degree patients and control groups (**A**) Forest plots of salivary s-IgA levels in high caries group and low caries group. (**B**) Forest plots of salivary s-IgA levels in high caries group and healthy control group. (**C**) Forest plots of salivary s-IgA levels in low caries group and healthy control group.

The results of the correlation between salivary s-IgA levels and the severity of dental caries were shown in Figure 5. Because of the limited number of studies on differences in the severity of caries (only nine studies were included) [21,25,5,33,36,38,39,41,46], and the heterogeneity in the studies was greater than 50% (*I*^2^>50%), a random-effects model was used (*P*<0.001). The results show that when comparing the salivary s-IgA levels of patients with high dental caries and patients with low dental caries (Figure 5A), we found that the salivary s-IgA levels of patients with high dental caries were significantly lower than those of patients with low dental caries (SMD = −0.89, 95%CI: [−1.46; −0.31], *P*=0.003). When comparing the salivary s-IgA levels of patients with high caries and healthy controls (Figure 5B), the salivary s-IgA levels of patients with high caries were significantly lower than those of healthy controls (SMD = −1.67, 95%CI: [−2.60; −0.74], *P*<0.001). When comparing the salivary s-IgA levels of patients with low caries and healthy controls (Figure 5C), the salivary s-IgA levels of patients with mild caries were significantly lower than those of healthy controls (SMD = −0.60, 95%CI: [−0.99; −0.20], *P*=0.003).

### Sensitivity analysis and publication bias

**Figure 6:**
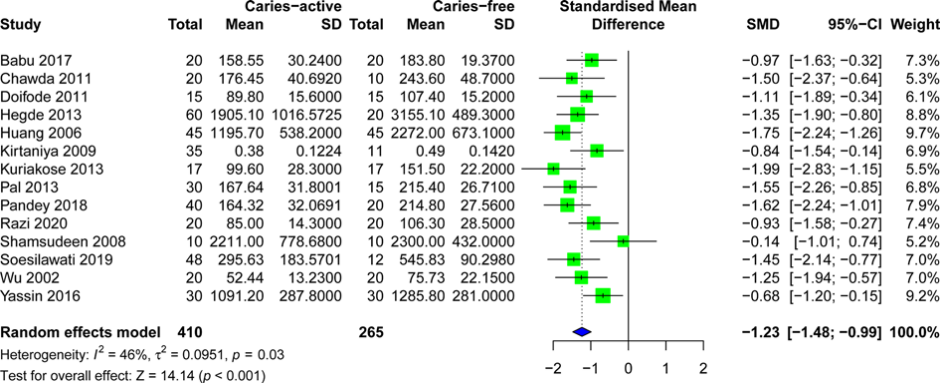
Forest plot for the salivary s-IgA levels between caries-active patients and healthy controls of 14 articles remained after sensitivity test

**Figure 7:**
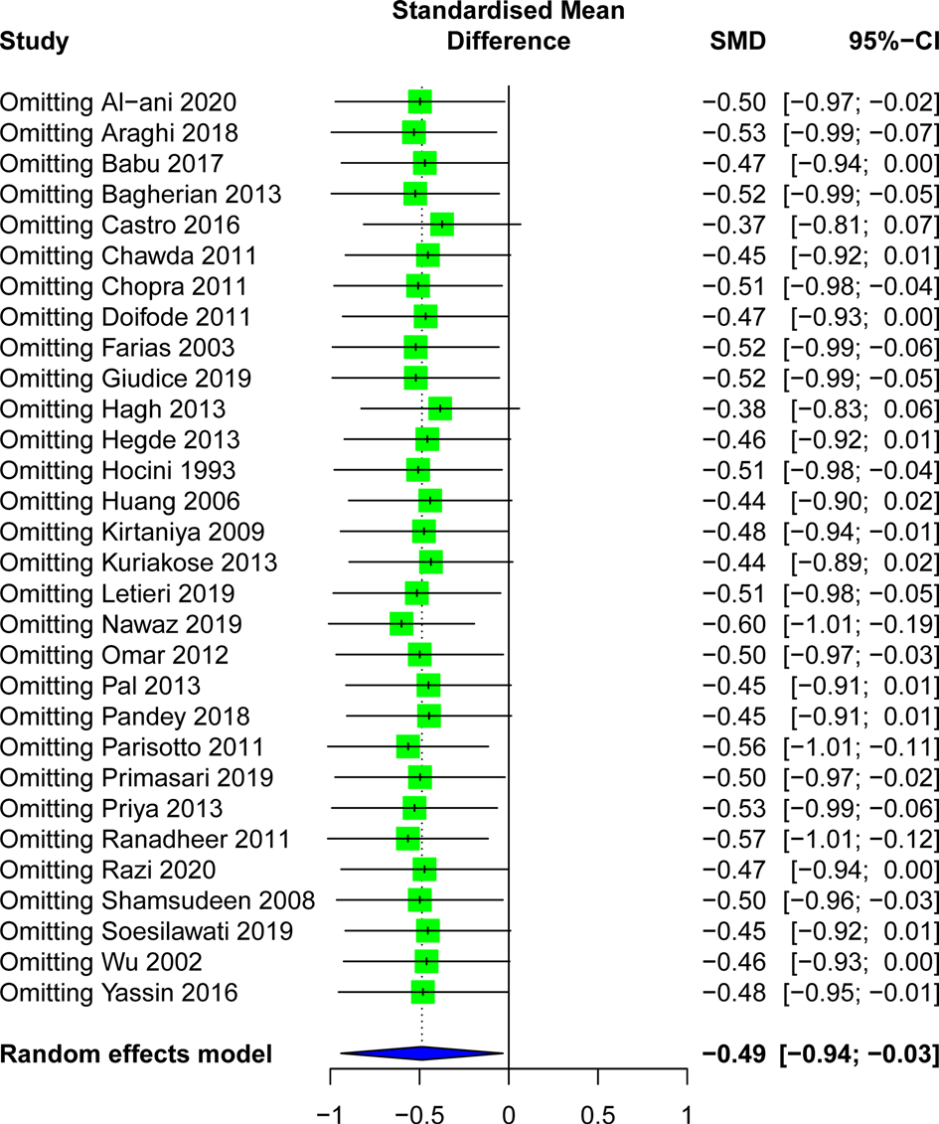
Sensitivity analyses for the association between salivary s-IgA levels and dental caries

**Figure 8:**
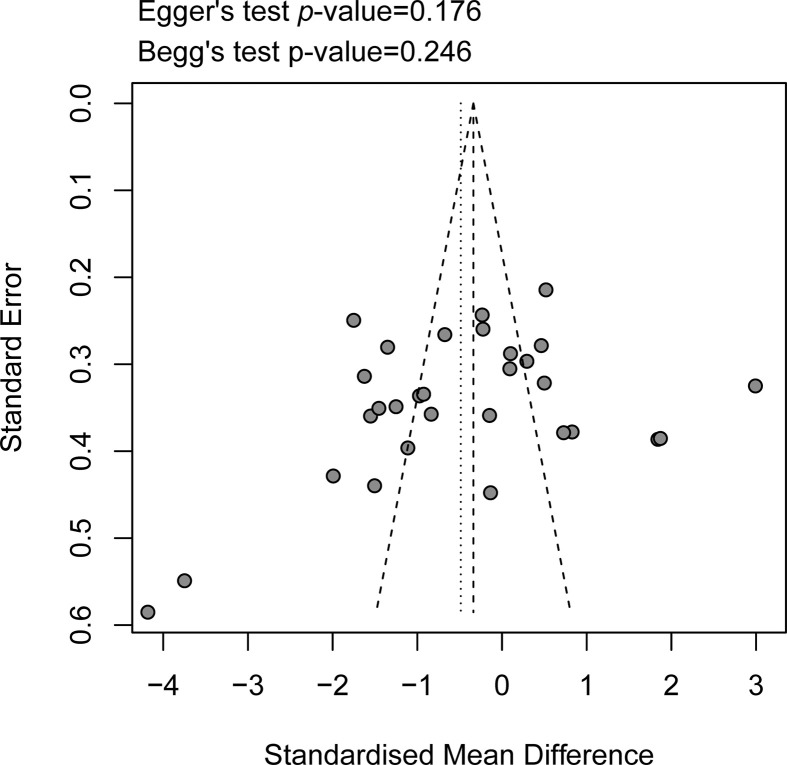
Assessment publication bias for the association between salivary s-IgA concentration and dental caries

A sensitivity analysis was performed to avoid heterogeneity. Figure 6 shows 14 studies with acceptable heterogeneity (*I*^2^ = 46%). The [22,25,27,5,32–34,38,39,44–48] caries-free healthy control group included 265 study subjects and the caries group included 410 study subjects, and the combined meta-analysis of caries activity was performed at lower levels of s-IgA (SMD = −1.23, 95%CI: [−1.48; −0.99], *P*<0.001). In the sensitivity analysis, the removal of any individual study did not change the overall statistical significance, indicating that the meta-analysis was relatively stable and reliable (Figure 7). Salivary s-IgA levels in caries patients and healthy controls were analyzed for publication bias. The magnitude of the effect was concentrated around the overall effect, with large samples concentrated at the top and small samples distributed around the bottom in an inverted funnel shape, indicating that publication bias had a small effect. The results showed that the effect of publication bias was small (Figure 8).

### Random-effects meta-regression analysis

Meta-regression analysis was used to find the source of heterogeneity. Age, dentition periods, region, and salivary s-IgA measurement type were used as covariates, but none of the factors showed significant confounding bias as a source of risk factors (Table 5).

**Table 5.** Meta-regression analysis coefficients for salivary s-IgA levels in the examined group of studies

Variables	Coefficient (SE)	95% Confidence interval	*P*
**Region**	−0.06 (0.37)	−0.83; 0.71	0.86
**Age**	−0.45 (1.09)	−2.71; 1.81	0.68
**Dentition Periods**	0.55 (0.63)	−0.74; 1.84	0.38
**Detection method**	−0.45 (0.44)	−1.35; 0.45	0.31

## Discussion

After reading the full text, according to the inclusion and exclusion criteria, 1 review, 1 republished study, 5 studies excluding caries-free control, and 26 studies that could not extract available data were excluded from this systematic evaluation. Finally, 30 case–control studies with 918 caries active patients and 627 healthy controls to explore the relationship between salivary s-IgA concentration and caries. About 16 of the studies reported lower levels of s-IgA in saliva from caries patients compared with healthy controls without caries, and 22 of the studies showed statistical differences. In general, our meta-analysis shows that the concentration of s-IgA in the saliva of caries patients was significantly lower than that of the normal control group (SMD = −0.49, 95%CI: [−0.94; −0.03], *P*=0.03), suggesting that that low concentration of saliva salivary s-IgA is associated with an increased risk of caries and may be used as a potential biomarker for screening caries susceptible population in the future.

Our results were inconsistent with previous meta-analysis results [12]. They found that the levels of salivary s-IgA in caries patients were significantly higher than those in healthy controls, and the increased salivary s-IgA levels were positively correlated with the occurrence of caries. However, the results of our study showed that the degree of caries activity was negatively correlated with salivary s-IgA concentration. Included in our meta-analysis, however, another 18 studies, including before 12 in previous meta-analysis study, two of them were not included in the present study. The reasons were as follows: Saliva is irritant [49]; lack of a control group [50]. So was excluded in our meta-analysis. We have extended the previous meta-analysis to show the correlation between salivary s-IgA levels and dental caries and conducted subgroup analysis, sensitivity analysis, and meta-regression analysis to find the source of heterogeneity.

The most important humoral antibody of oral mucosal specific immunity is s-IgA [51,52]. One typical s-IgA is mainly composed of two IgA monomers (i.e. dimer IgA), one J chain and one secretory component (SC). S-IgA and J chains are synthesized by mucosal epithelial lamina propria or salivary gland plasma cells, and SC, as dimer IgA specific receptor, is located on the epithelial cell membrane and synthesized by epithelial cells. When IgA binds SC segments, its structure is more compact and is not easy to be enzymatically hydrolyzed, which is conducive to the high antibody activity of IgA on the mucosal surface and in the exocrine secretion [53]. The main function of s-IgA is to block or prevent pathogenic bacteria from adhering to the mucosal epithelial cells or the surface of teeth. S-IgA cannot activate complement but can activate the complement system by the alternative pathway in the polymerization state. S-IgA has no bactericidal effect and the effect of promoting the formation of tonic complement fragment and lacks a direct conditioning effect, but IgA-mediated antibody-dependent cytotoxicity can be found in the human body [7].

Salivary s-IgA is a relatively stable anti-inflammatory immunoglobulin, which can maintain its activity in the environment of oral proteolytic enzymes for a long time [54,55]. The potential link between caries and saliva s-IgA levels remains to be elaborated. One possible mechanism is that saliva s-IgA prevents pathogens from adhering to the tooth surface. Salivary s-IgA inhibits microbial colonization and neutralizes microbial enzymes or toxins [56–58]. Salivary s-IgA antibody can prevent the colonization of *Streptococcus mutans* by neutralizing glycosyltransferase (GTF), thus reducing the rate of GTF binding to *Streptococcus mutans* pili, thus inhibiting the development of dental caries [59,60]. Another potential mechanism is that salivary s-IgA can synergize and promote the bacteriostasis of the lactoferrin and peroxidase system, which can also explain the correlation between salivary s-IgA and caries [61–63].

During the occurrence and development of dental caries, the host’s immune defense response is also very important. Most of the people included in the present study were children (1053). The decrease of s-IgA in the saliva of children with dental caries may be due to the challenge of various microorganisms in the oral cavity in their childhood. Their immune system or lymphatic system is developing or immature, and they will swallow saliva repeatedly, resulting in s-IgA decreased [33]. Besides, the feeding method of the child and the level of s-IgA in the mother’s saliva and breast milk will also affect the level of s-IgA in the oral saliva [64]. In the formation of dental caries, cariogenic microorganisms need to overcome the host’s nonspecific defense barriers (cleaning mechanism, swallowing and saliva flow, etc.), and then must escape the recognition of soluble immune or nonimmune host molecules in host secretions [7]. The above processes were all related to the formation of dental caries. Salivary s-IgA can bind to the surface antigens of microorganisms in saliva to make them agglutinate, thereby promoting their rapid elimination and preventing the occurrence of dental caries [10]. However, some studies have suggested that microorganisms can protect themselves from host immune attack by forming biofilms and reducing the expression of antigens [65,66], which will cause higher levels of s-IgA in the saliva of healthy people than patients with caries. Dental caries was caused by the imbalance between enamel demineralization induced by bacterial biofilms and oral defenses including immune and inflammatory responses. The level of s-IgA in the saliva of caries patients was significantly lower than that of healthy people, but the activation mode of salivary s-IgA and the protective mechanism against caries still need further study.

The results of Primasari et al. [41] and Al-ani et al. [20] found that there was no difference in salivary s-IgA levels between caries-free people and caries patients, which was contradictory with the results of the present study. The results obtained by the above studies may be due to the small sample size and different criteria for judging dental caries. Besides, it is recommended that salivary s-IgA levels be measured in children over the age of 6, whose immune system is considered complete [46]. Jafarzadeh et al. [67] found that salivary s-IgA levels increase with age before the age of 60, and slightly decrease in the 61–70 age group. The reasons described above may cause conflicting results with this study. Fidalgo et al [12] systematic review and meta-analysis have reported the opposite results. First, it may be because the saliva requirements were not selected. They included contains both nonirritating saliva and irritating saliva. The current commonly used saliva source has irritant parotid gland fluid, irritant whole saliva, and nonirritating whole saliva. Nonirritating whole saliva is more reasonable to analyze because the interaction between antibacterial substances and bacteria in the saliva is mostly occurred in nonirritating whole saliva [68]. Also, subjects’ emotions, inflammation, infection, systemic diseases, medication, age, and the interaction of various substances in saliva can all affect the experimental results, and the above factors should be avoided as far as possible. This was why the present study only included articles that collected nonirritating saliva.

Although the results of most studies included in the present study indicate that the saliva s-IgA concentration of caries patients is significantly lower than that of the normal control group. Considering other factors that may influence the relationship between salivary s-IgA levels and dental caries, we conducted subgroup analysis based on age, region, type of dentition, and salivary s-IgA detection method. Subgroup analysis revealed a more consistent association between salivary s-IgA levels and caries in children. Salivary s-IgA levels tended to be lower in children's caries patients than in healthy controls, but the heterogeneity of the results was significant. This also reminds us that it may be that children were usually unable to maintain oral health properly and were one of the most vulnerable to other oral diseases. In addition to age, other important factors could affect the levels of s-IgA in saliva, including saliva flow rate, smoking, pregnancy, and other stress factors [7,69,70]. Therefore, the level of saliva s-IgA is of great importance to the prevention of dental caries in children. Studies on different regions showed that the correlation between the levels of salivary s-IgA in Asian caries patients was more significant than in other regions. It may be related to the economic level, social background, and dietary factors. Studies in other regions did not show relevance, possibly due to the small number of studies and subjects included, and the inconsistent criteria for determining dental caries. We have made correction according to the reviewer’s comments. Besides, the saliva s-IgA level of dental caries patients in the mixed dentition group compared with the primary dentition and permanent dentition group was different from that of the control group, but the difference was not statistically significant (*P*=0.06). This was consistent with what we have previously obtained in the subgroup of different age patients. It further shows that salivary s-IgA concentration has a better correlation in children. Furthermore, other potential confounders were analyzed by using meta-regression, but none of them were related to salivary s-IgA levels, suggesting that these factors were unlikely to explain the differences in salivary s-IgA levels between caries patients and healthy controls.

The salivary s-IgA detection method also results in some differences [61]. At present, the commonly used methods for the determination of s-IgA concentration in saliva include immunoturbidimetry, RIA, and ELISA. The accuracy of the immunoturbidimetric method is poor and the error of manual measurement is large; RIA is time-consuming and requires the use of radioisotopes but is highly sensitive. The NABA method uses a biotin-labeled antibody to replace the enzyme-labeled antibody in the ordinary ELISA method. Due to the higher affinity between biotin and avidin, the stability of the experiment is improved and the reaction time is shortened. However, the operation process is more complicated and is used less. ELISA is the leading technology in clinical immunoassays with specificity, sensitivity, ease of operation, and stability of reagents. More importantly, it does not pose a threat to the environment. Although the salivary s-IgA assay used was not an exclusion criterion, the included major studies used confidence methods to evaluate s-IgA. All included studies indicated the use of commercial kits under the manufacturer’s instructions.

We further included the gender grouping of the four studies and found that in both the male group and the female group, the s-IgA concentration in the saliva of caries patients was significantly lower than that of people without caries. The relationship between gender and salivary s-IgA levels was rarely reported. The results of the present study also showed that gender did not affect on the difference in saliva s-IgA levels between caries patients and healthy controls. Similarly, the nine included studies were grouped according to the degree of caries, and the results showed that the salivary s-IgA concentration of patients with high caries was significantly lower than that of patients with low caries, and the salivary s-IgA concentration of patients with high caries was significantly lower than that of the caries-free group. The salivary s-IgA concentration of patients with low caries was significantly lower than that of the caries-free group. This indicates that the concentration of salivary s-IgA was related to the degree of caries, and the concentration of salivary s-IgA was also dose-related with the severity of caries.

Of course, our meta-analysis also has some limitations. Due to language limitations, our study was unable to include all available data covering salivary s-IgA and caries. The included studies were case–control studies, demonstrating a possible association between salivary s-IgA and caries, but not a causal relationship between salivary s-IgA and caries. Other types of study designs have yet to prove whether low salivary s-IgA levels play a role in the development of caries, or whether caries induces low salivary s-IgA expression in the population. Our adjustment for the potential confounders included in the study was limited. Although we used the subgroup analysis and meta-regression analysis to find the source of the heterogeneity, other sources of heterogeneity may still affect the results, such as saliva flow rate, smoking, pregnancy, and body fat composition and other factors, such as stress can affect the accuracy of the results. But there were a few research reports on those clinical data, so no meta-regression analysis was conducted.

The conclusions are drawn from this meta-analysis still need us to further verify the specific protective mechanism of s-IgA against caries in animal or cell experiments. Using the protective effect of saliva s-IgA, we can further develop vaccines to prevent caries. In susceptible people, the body is induced to produce specific and non-specific s-IgA, and the content of s-IgA in the saliva is increased, thereby achieving the effect of preventing dental caries [71]. It is also possible to make a protective agent containing s-IgA and apply it on the surface of the teeth of patients with a high incidence of dental caries, or to use mouthwash or toothpaste containing s-IgA to weaken or eliminate potential cariogenic factors in a local area. Besides, the study of Choonharuangdej et al. [72] showed that the group with higher IgA concentration in gingival crevicular fluid had less severe periodontitis. Therefore, the concentration of GCF-s-IgA antibody may also be related to the occurrence and development of periodontitis. The research on s-IgA and endodontic and periodontal pathologies is also an important direction worthy of our further research and exploration.

## Conclusion

In summary, the present study showed that the levels of salivary s-IgA in caries patients were lower than that in healthy control group, indicating that the decrease of salivary s-IgA levels was closely related to the progress of caries. The levels of salivary s-IgA can be used as a valuable biomarker to evaluate the clinical status of caries patients. To confirm this finding and determine whether salivary s-IgA has clinical value in the treatment of caries patients, larger studies and better study designs are needed in the future.

## Supplementary Material

Supplementary Figures S1-S4 and Tables S1-S2

## Data Availability

The data used to support the findings of this study are included within the article.

## Abbreviations

ELISA: enzyme-linked immunosorbent assay
NABA: noncompetitive avidin-biotin immunoenzymatic assay
NOS: Newcastle–Ottawa scale
RIA: radioimmunoassay
SMD: standardized mean difference
