# Supplementary material for: Association between salivary s-IgA concentration and dental caries: an updated meta-analysis
Source: Biosci Rep. 2020 Dec 22;40(12):BSR20203208. doi: 10.1042/BSR20203208 (PMC7755122; doi:10.1042/BSR20203208)
Supplement: Supplementary Figures S1-S4 and Tables S1-S2 [file BSR-2020-3208_supp.pdf]

Supplementary Figures:

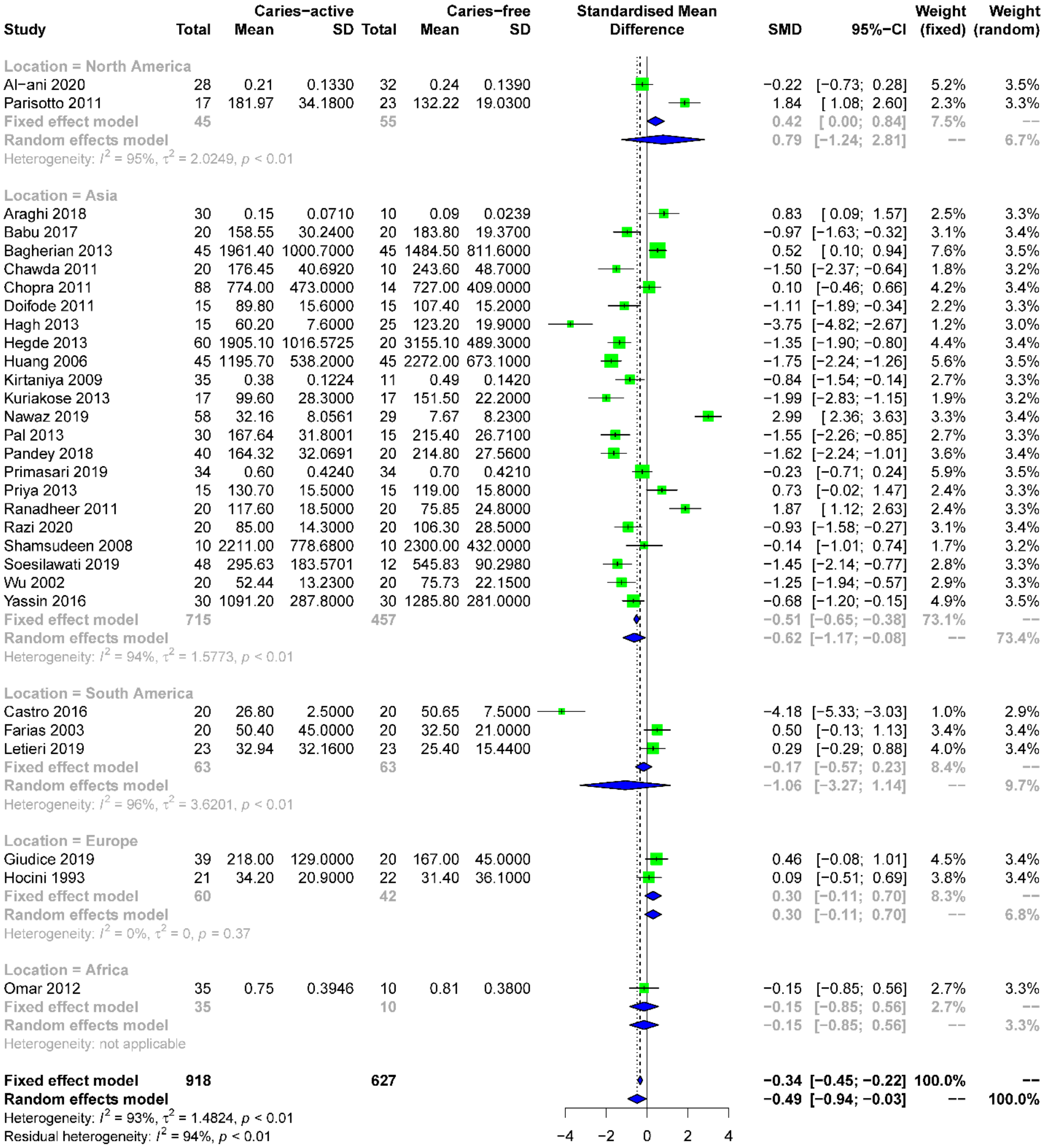

Figure S1: Subgroup analysis for the differences of salivary s-IgA levels between caries patients and healthy controls in different regions.

Study	Caries−active Total	Caries−active Mean	Caries−active SD	Caries−free Total	Caries−free Mean	Caries−free SD	SMD	95%−CI	Weight (fixed)	Weight (random)
**Age ≥ 18**										
Al−ani 2020	28	0.21	0.1330	32	0.24	0.1390	−0.22	[−0.73; 0.28]	5.2%	3.5%
Araghi 2018	30	0.15	0.0710	10	0.09	0.0239	0.83	[0.09; 1.57]	2.5%	3.3%
Castro 2016	20	26.80	2.5000	20	50.65	7.5000	−4.18	[−5.33; −3.03]	1.0%	2.9%
Chopra 2011	88	774.00	473.0000	14	727.00	409.0000	0.10	[−0.46; 0.66]	4.2%	3.4%
Hagh 2013	15	60.20	7.6000	25	123.20	19.9000	−3.75	[−4.82; −2.67]	1.2%	3.0%
Hegde 2013	60	1905.10	1016.5725	20	3155.10	489.3000	−1.35	[−1.90; −0.80]	4.4%	3.4%
Hocini 1993	21	34.20	20.9000	22	31.40	36.1000	0.09	[−0.51; 0.69]	3.8%	3.4%
Nawaz 2019	58	32.16	8.0561	29	7.67	8.2300	2.99	[2.36; 3.63]	3.3%	3.4%
Fixed effect model	320			172			−0.12	[−0.35; 0.11]	25.6%	--
Random effects model							−0.64	[−1.92; 0.64]	--	26.3%
Heterogeneity: $I^2 = 97\%$, $\tau^2 = 3.2744$, $p < 0.01$										
**Age < 18**										
Babu 2017	20	158.55	30.2400	20	183.80	19.3700	−0.97	[−1.63; −0.32]	3.1%	3.4%
Bagherian 2013	45	1961.40	1000.7000	45	1484.50	811.6000	0.52	[0.10; 0.94]	7.6%	3.5%
Chawda 2011	20	176.45	40.6920	10	243.60	48.7000	−1.50	[−2.37; −0.64]	1.8%	3.2%
Doifode 2011	15	89.80	15.6000	15	107.40	15.2000	−1.11	[−1.89; −0.34]	2.2%	3.3%
Farias 2003	20	50.40	45.0000	20	32.50	21.0000	0.50	[−0.13; 1.13]	3.4%	3.4%
Giudice 2019	39	218.00	129.0000	20	167.00	45.0000	0.46	[−0.08; 1.01]	4.5%	3.4%
Huang 2006	45	1195.70	538.2000	45	2272.00	673.1000	−1.75	[−2.24; −1.26]	5.6%	3.5%
Kirtaniya 2009	35	0.38	0.1224	11	0.49	0.1420	−0.84	[−1.54; −0.14]	2.7%	3.3%
Kuriakose 2013	17	99.60	28.3000	17	151.50	22.2000	−1.99	[−2.83; −1.15]	1.9%	3.2%
Letieri 2019	23	32.94	32.1600	23	25.40	15.4400	0.29	[−0.29; 0.88]	4.0%	3.4%
Omar 2012	35	0.75	0.3946	10	0.81	0.3800	−0.15	[−0.85; 0.56]	2.7%	3.3%
Pal 2013	30	167.64	31.8001	15	215.40	26.7100	−1.55	[−2.26; −0.85]	2.7%	3.3%
Pandey 2018	40	164.32	32.0691	20	214.80	27.5600	−1.62	[−2.24; −1.01]	3.6%	3.4%
Parisotto 2011	17	181.97	34.1800	23	132.22	19.0300	1.84	[1.08; 2.60]	2.3%	3.3%
Primasari 2019	34	0.60	0.4240	34	0.70	0.4210	−0.23	[−0.71; 0.24]	5.9%	3.5%
Priya 2013	15	130.70	15.5000	15	119.00	15.8000	0.73	[−0.02; 1.47]	2.4%	3.3%
Ranadheer 2011	20	117.60	18.5000	20	75.85	24.8000	1.87	[1.12; 2.63]	2.4%	3.3%
Razi 2020	20	85.00	14.3000	20	106.30	28.5000	−0.93	[−1.58; −0.27]	3.1%	3.4%
Shamsudeen 2008	10	2211.00	778.6800	10	2300.00	432.0000	−0.14	[−1.01; 0.74]	1.7%	3.2%
Soesilawati 2019	48	295.63	183.5701	12	545.83	90.2980	−1.45	[−2.14; −0.77]	2.8%	3.3%
Wu 2002	20	52.44	13.2300	20	75.73	22.1500	−1.25	[−1.94; −0.57]	2.9%	3.3%
Yassin 2016	30	1091.20	287.8000	30	1285.80	281.0000	−0.68	[−1.20; −0.15]	4.9%	3.5%
Fixed effect model	598			455			−0.41	[−0.55; −0.28]	74.4%	--
Random effects model							−0.45	[−0.89; −0.01]	--	73.7%
Heterogeneity: $I^2 = 90\%$, $\tau^2 = 0.9931$, $p < 0.01$										
**Fixed effect model**	**918**			**627**			**−0.34**	**[−0.45; −0.22]**	**100.0%**	**--**
**Random effects model**							**−0.49**	**[−0.94; −0.03]**	**--**	**100.0%**
Heterogeneity: $I^2 = 93\%$, $\tau^2 = 1.4824$, $p < 0.01$										
Residual heterogeneity: $I^2 = 94\%$, $p < 0.01$										

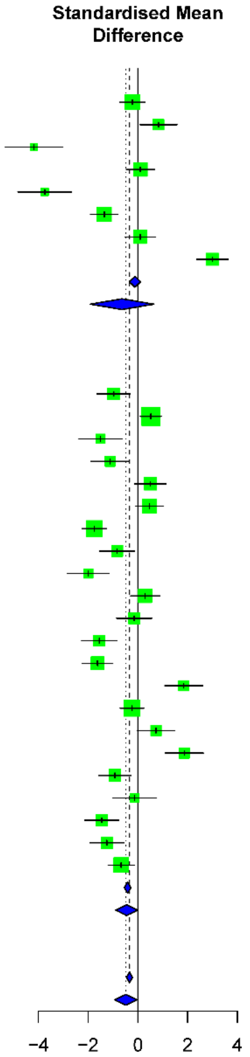

Figure S2: Subgroup analysis for the differences of salivary s-IgA levels between caries patients and healthy controls in different ages.

Study	Caries−active Total	Caries−active Mean	Caries−active SD	Caries−free Total	Caries−free Mean	Caries−free SD	SMD	95%−CI	Weight (fixed)	Weight (random)
**Dentition = Permanent dentition**										
Al−ani 2020	28	0.21	0.1330	32	0.24	0.1390	−0.22	[−0.73; 0.28]	5.2%	3.5%
Araghi 2018	30	0.15	0.0710	10	0.09	0.0239	0.83	[0.09; 1.57]	2.5%	3.3%
Castro 2016	20	26.80	2.5000	20	50.65	7.5000	−4.18	[−5.33; −3.03]	1.0%	2.9%
Chopra 2011	88	774.00	473.0000	14	727.00	409.0000	0.10	[−0.46; 0.66]	4.2%	3.4%
Hagh 2013	15	60.20	7.6000	25	123.20	19.9000	−3.75	[−4.82; −2.67]	1.2%	3.0%
Hegde 2013	60	1905.10	1016.5725	20	3155.10	489.3000	−1.35	[−1.90; −0.80]	4.4%	3.4%
Hocini 1993	21	34.20	20.9000	22	31.40	36.1000	0.09	[−0.51; 0.69]	3.8%	3.4%
Nawaz 2019	58	32.16	8.0561	29	7.67	8.2300	2.99	[2.36; 3.63]	3.3%	3.4%
Razi 2020	20	85.00	14.3000	20	106.30	28.5000	−0.93	[−1.58; −0.27]	3.1%	3.4%
Fixed effect model	340			192			−0.21	[−0.42; 0.01]	28.7%	--
Random effects model							−0.66	[−1.81; 0.48]	--	29.7%
Heterogeneity: $I^2 = 96\%$, $\tau^2 = 2.9119$, $p < 0.01$										
**Dentition = Mixed dentition**										
Babu 2017	20	158.55	30.2400	20	183.80	19.3700	−0.97	[−1.63; −0.32]	3.1%	3.4%
Chawda 2011	20	176.45	40.6920	10	243.60	48.7000	−1.50	[−2.37; −0.64]	1.8%	3.2%
Doifode 2011	15	89.80	15.6000	15	107.40	15.2000	−1.11	[−1.89; −0.34]	2.2%	3.3%
Giudice 2019	39	218.00	129.0000	20	167.00	45.0000	0.46	[−0.08; 1.01]	4.5%	3.4%
Kirtaniya 2009	35	0.38	0.1224	11	0.49	0.1420	−0.84	[−1.54; −0.14]	2.7%	3.3%
Pal 2013	30	167.64	31.8001	15	215.40	26.7100	−1.55	[−2.26; −0.85]	2.7%	3.3%
Pandey 2018	40	164.32	32.0691	20	214.80	27.5600	−1.62	[−2.24; −1.01]	3.6%	3.4%
Priya 2013	15	130.70	15.5000	15	119.00	15.8000	0.73	[−0.02; 1.47]	2.4%	3.3%
Ranadheer 2011	20	117.60	18.5000	20	75.85	24.8000	1.87	[1.12; 2.63]	2.4%	3.3%
Soesilawati 2019	48	295.63	183.5701	12	545.83	90.2980	−1.45	[−2.14; −0.77]	2.8%	3.3%
Yassin 2016	30	1091.20	287.8000	30	1285.80	281.0000	−0.68	[−1.20; −0.15]	4.9%	3.5%
Fixed effect model	312			188			−0.59	[−0.79; −0.39]	33.2%	--
Random effects model							−0.61	[−1.24; 0.03]	--	36.7%
Heterogeneity: $I^2 = 90\%$, $\tau^2 = 1.0432$, $p < 0.01$										
**Dentition = Deciduous dentition**										
Bagherian 2013	45	1961.40	1000.7000	45	1484.50	811.6000	0.52	[0.10; 0.94]	7.6%	3.5%
Farias 2003	20	50.40	45.0000	20	32.50	21.0000	0.50	[−0.13; 1.13]	3.4%	3.4%
Huang 2006	45	1195.70	538.2000	45	2272.00	673.1000	−1.75	[−2.24; −1.26]	5.6%	3.5%
Kuriakose 2013	17	99.60	28.3000	17	151.50	22.2000	−1.99	[−2.83; −1.15]	1.9%	3.2%
Letieri 2019	23	32.94	32.1600	23	25.40	15.4400	0.29	[−0.29; 0.88]	4.0%	3.4%
Omar 2012	35	0.75	0.3946	10	0.81	0.3800	−0.15	[−0.85; 0.56]	2.7%	3.3%
Parisotto 2011	17	181.97	34.1800	23	132.22	19.0300	1.84	[1.08; 2.60]	2.3%	3.3%
Primasari 2019	34	0.60	0.4240	34	0.70	0.4210	−0.23	[−0.71; 0.24]	5.9%	3.5%
Shamsudeen 2008	10	2211.00	778.6800	10	2300.00	432.0000	−0.14	[−1.01; 0.74]	1.7%	3.2%
Wu 2002	20	52.44	13.2300	20	75.73	22.1500	−1.25	[−1.94; −0.57]	2.9%	3.3%
Fixed effect model	266			247			−0.21	[−0.40; −0.03]	38.1%	--
Random effects model							−0.23	[−0.91; 0.44]	--	33.6%
Heterogeneity: $I^2 = 92\%$, $\tau^2 = 1.0791$, $p < 0.01$										
**Fixed effect model**	**918**			**627**			**−0.34**	**[−0.45; −0.22]**	**100.0%**	**--**
**Random effects model**							**−0.49**	**[−0.94; −0.03]**	**--**	**100.0%**
Heterogeneity: $I^2 = 93\%$, $\tau^2 = 1.4824$, $p < 0.01$										
Residual heterogeneity: $I^2 = 94\%$, $p < 0.01$										

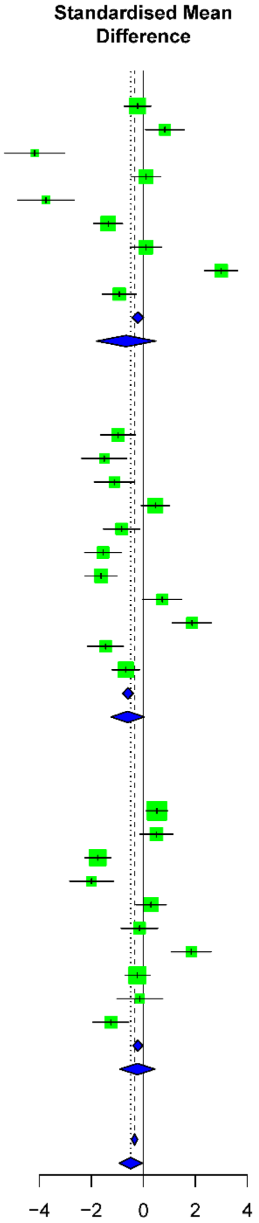

Figure S3: Subgroup analysis for the differences of salivary s-IgA levels between caries patients and healthy controls in different type of dentitions.

Study	Caries−active Total	Caries−active Mean	Caries−active SD	Caries−free Total	Caries−free Mean	Caries−free SD	SMD	95%−CI	Weight (fixed)	Weight (random)
**Detection_method = ELISA**										
Al−ani 2020	28	0.21	0.1330	32	0.24	0.1390	−0.22	[−0.73; 0.28]	5.2%	3.5%
Babu 2017	20	158.55	30.2400	20	183.80	19.3700	−0.97	[−1.63; −0.32]	3.1%	3.4%
Bagherian 2013	45	1961.40	1000.7000	45	1484.50	811.6000	0.52	[0.10; 0.94]	7.6%	3.5%
Castro 2016	20	26.80	2.5000	20	50.65	7.5000	−4.18	[−5.33; −3.03]	1.0%	2.9%
Chopra 2011	88	774.00	473.0000	14	727.00	409.0000	0.10	[−0.46; 0.66]	4.2%	3.4%
Giudice 2019	39	218.00	129.0000	20	167.00	45.0000	0.46	[−0.08; 1.01]	4.5%	3.4%
Hagh 2013	15	60.20	7.6000	25	123.20	19.9000	−3.75	[−4.82; −2.67]	1.2%	3.0%
Hocini 1993	21	34.20	20.9000	22	31.40	36.1000	0.09	[−0.51; 0.69]	3.8%	3.4%
Kirtaniya 2009	35	0.38	0.1224	11	0.49	0.1420	−0.84	[−1.54; −0.14]	2.7%	3.3%
Kuriakose 2013	17	99.60	28.3000	17	151.50	22.2000	−1.99	[−2.83; −1.15]	1.9%	3.2%
Letieri 2019	23	32.94	32.1600	23	25.40	15.4400	0.29	[−0.29; 0.88]	4.0%	3.4%
Nawaz 2019	58	32.16	8.0561	29	7.67	8.2300	2.99	[2.36; 3.63]	3.3%	3.4%
Omar 2012	35	0.75	0.3946	10	0.81	0.3800	−0.15	[−0.85; 0.56]	2.7%	3.3%
Pal 2013	30	167.64	31.8001	15	215.40	26.7100	−1.55	[−2.26; −0.85]	2.7%	3.3%
Pandey 2018	40	164.32	32.0691	20	214.80	27.5600	−1.62	[−2.24; −1.01]	3.6%	3.4%
Parisotto 2011	17	181.97	34.1800	23	132.22	19.0300	1.84	[1.08; 2.60]	2.3%	3.3%
Primasari 2019	34	0.60	0.4240	34	0.70	0.4210	−0.23	[−0.71; 0.24]	5.9%	3.5%
Priya 2013	15	130.70	15.5000	15	119.00	15.8000	0.73	[−0.02; 1.47]	2.4%	3.3%
Ranadheer 2011	20	117.60	18.5000	20	75.85	24.8000	1.87	[1.12; 2.63]	2.4%	3.3%
Razi 2020	20	85.00	14.3000	20	106.30	28.5000	−0.93	[−1.58; −0.27]	3.1%	3.4%
Soesilawati 2019	48	295.63	183.5701	12	545.83	90.2980	−1.45	[−2.14; −0.77]	2.8%	3.3%
**Fixed effect model**	668			447			−0.13	[−0.27; 0.01]	70.5%	--
**Random effects model**							−0.39	[−0.97; 0.19]	--	70.0%
Heterogeneity: $I^2 = 94\%$, $\tau^2 = 1.7181$, $p < 0.01$										
**Detection_method = Immunoturbidimetry**										
Araghi 2018	30	0.15	0.0710	10	0.09	0.0239	0.83	[0.09; 1.57]	2.5%	3.3%
Chawda 2011	20	176.45	40.6920	10	243.60	48.7000	−1.50	[−2.37; −0.64]	1.8%	3.2%
Doifode 2011	15	89.80	15.6000	15	107.40	15.2000	−1.11	[−1.89; −0.34]	2.2%	3.3%
Farias 2003	20	50.40	45.0000	20	32.50	21.0000	0.50	[−0.13; 1.13]	3.4%	3.4%
Hegde 2013	60	1905.10	1016.5725	20	3155.10	489.3000	−1.35	[−1.90; −0.80]	4.4%	3.4%
Shamsudeen 2008	10	2211.00	778.6800	10	2300.00	432.0000	−0.14	[−1.01; 0.74]	1.7%	3.2%
Yassin 2016	30	1091.20	287.8000	30	1285.80	281.0000	−0.68	[−1.20; −0.15]	4.9%	3.5%
**Fixed effect model**	185			115			−0.53	[−0.78; −0.27]	21.0%	--
**Random effects model**							−0.49	[−1.16; 0.17]	--	23.2%
Heterogeneity: $I^2 = 85\%$, $\tau^2 = 0.6756$, $p < 0.01$										
**Detection_method = RIA**										
Huang 2006	45	1195.70	538.2000	45	2272.00	673.1000	−1.75	[−2.24; −1.26]	5.6%	3.5%
**Fixed effect model**	45			45			−1.75	[−2.24; −1.26]	5.6%	--
**Random effects model**							−1.75	[−2.24; −1.26]	--	3.5%
Heterogeneity: not applicable										
**Detection_method = NABA**										
Wu 2002	20	52.44	13.2300	20	75.73	22.1500	−1.25	[−1.94; −0.57]	2.9%	3.3%
**Fixed effect model**	20			20			−1.25	[−1.94; −0.57]	2.9%	--
**Random effects model**							−1.25	[−1.94; −0.57]	--	3.3%
Heterogeneity: not applicable										
**Fixed effect model**	**918**			**627**			**−0.34**	**[−0.45; −0.22]**	**100.0%**	**--**
**Random effects model**							**−0.49**	**[−0.94; −0.03]**	**--**	**100.0%**
Heterogeneity: $I^2 = 93\%$, $\tau^2 = 1.4824$, $p < 0.01$										
Residual heterogeneity: $I^2 = 93\%$, $p < 0.01$										

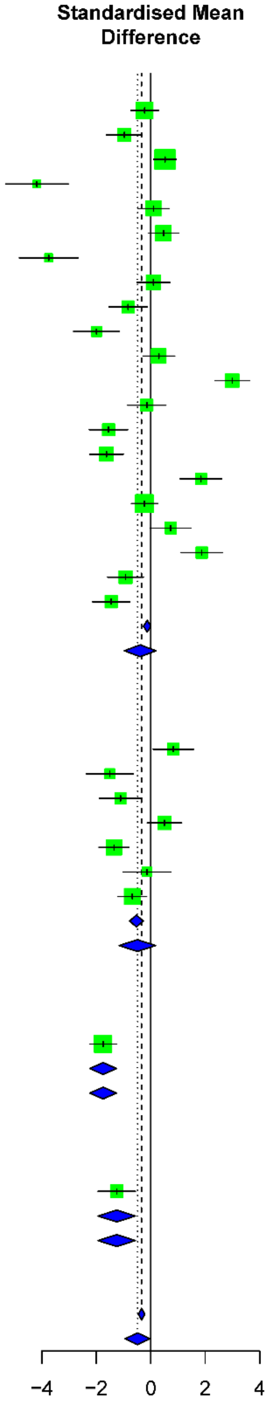

Figure S4: Subgroup analysis for the differences of salivary s-IgA levels between caries patients and healthy controls in different detection methods.

PRISMA 2009 Checklist

Section/topic	#	Checklist item	Reported on page #
**TITLE**			
Title	1	Identify the report as a systematic review, meta-analysis, or both.	Page 1
**ABSTRACT**			
Structured summary	2	Provide a structured summary including, as applicable: background; objectives; data sources; study eligibility criteria, participants, and interventions; study appraisal and synthesis methods; results; limitations; conclusions and implications of key findings; systematic review registration number.	Page 2
**INTRODUCTION**			
Rationale	3	Describe the rationale for the review in the context of what is already known.	Page 3
Objectives	4	Provide an explicit statement of questions being addressed with reference to participants, interventions, comparisons, outcomes, and study design (PICOS).	Page 4
**METHODS**			
Protocol and registration	5	Indicate if a review protocol exists, if and where it can be accessed (e.g., Web address), and, if available, provide registration information including registration number.	Page 4
Eligibility criteria	6	Specify study characteristics (e.g., PICOS, length of follow-up) and report characteristics (e.g., years considered, language, publication status) used as criteria for eligibility, giving rationale.	Page 5
Information sources	7	Describe all information sources (e.g., databases with dates of coverage, contact with study authors to identify additional studies) in the search and date last searched.	Page 5
Search	8	Present full electronic search strategy for at least one database, including any limits used, such that it could be repeated.	Page 5
Study selection	9	State the process for selecting studies (i.e., screening, eligibility, included in systematic review, and, if applicable, included in the meta-analysis).	Page 5-6
Data collection process	10	Describe method of data extraction from reports (e.g., piloted forms, independently, in duplicate) and any processes for obtaining and confirming data from investigators.	Page 5-6
Data items	11	List and define all variables for which data were sought (e.g., PICOS, funding sources) and any assumptions and simplifications made.	Page 5-6
Risk of bias in individual studies	12	Describe methods used for assessing risk of bias of individual studies (including specification of whether this was done at the study or outcome level), and how this information is to be used in any data synthesis.	Page 5-6
Summary measures	13	State the principal summary measures (e.g., risk ratio, difference in means).	Page 6-7
Synthesis of results	14	Describe the methods of handling data and combining results of studies, if done, including measures of consistency (e.g., I^2) for each meta-analysis.	Page 7

PRISMA 2009 Checklist

Section/topic	#	Checklist item	Reported on page #
Risk of bias across studies	15	Specify any assessment of risk of bias that may affect the cumulative evidence (e.g., publication bias, selective reporting within studies).	Page 7
Additional analyses	16	Describe methods of additional analyses (e.g., sensitivity or subgroup analyses, meta-regression), if done, indicating which were pre-specified.	Page 7
**RESULTS**			
Study selection	17	Give numbers of studies screened, assessed for eligibility, and included in the review, with reasons for exclusions at each stage, ideally with a flow diagram.	Page 8
Study characteristics	18	For each study, present characteristics for which data were extracted (e.g., study size, PICOS, follow-up period) and provide the citations.	Page 8
Risk of bias within studies	19	Present data on risk of bias of each study and, if available, any outcome level assessment (see item 12).	Page 8-11
Results of individual studies	20	For all outcomes considered (benefits or harms), present, for each study: (a) simple summary data for each intervention group (b) effect estimates and confidence intervals, ideally with a forest plot.	Page 8-11
Synthesis of results	21	Present results of each meta-analysis done, including confidence intervals and measures of consistency.	Page 8-11
Risk of bias across studies	22	Present results of any assessment of risk of bias across studies (see Item 15).	Page 8-11
Additional analysis	23	Give results of additional analyses, if done (e.g., sensitivity or subgroup analyses, meta-regression [see Item 16]).	Page 11-12
**DISCUSSION**			
Summary of evidence	24	Summarize the main findings including the strength of evidence for each main outcome; consider their relevance to key groups (e.g., healthcare providers, users, and policy makers).	Page 12-17
Limitations	25	Discuss limitations at study and outcome level (e.g., risk of bias), and at review-level (e.g., incomplete retrieval of identified research, reporting bias).	Page 17-18
Conclusions	26	Provide a general interpretation of the results in the context of other evidence, and implications for future research.	Page 18
**FUNDING**			
Funding	27	Describe sources of funding for the systematic review and other support (e.g., supply of data); role of funders for the systematic review.	Page 19

From: Moher D, Liberati A, Tetzlaff J, Altman DG, The PRISMA Group (2009). Preferred Reporting Items for Systematic Reviews and Meta-Analyses: The PRISMA Statement. PLoS Med 6(7): e1000097. doi:10.1371/journal.pmed1000097

For more information, visit: **www.prisma-statement.org**.

Table S2. Newcastle - Ottawa Quality Assessment Scale for case control studies
Website: http://www.ohri.ca/programs/clinical_epidemiology/oxford.asp

Note: A study can be awarded a maximum of one star for each numbered item within the Selection and Exposure categories. A maximum of two stars can be given for Comparability.

Item	Stars

Selection

1) Is the case definition adequate?
   a) yes, with independent validation □✵
   b) yes, e.g. record linkage or based on self-reports
   c) no description

2) Representativeness of the cases
   a) consecutive or obviously representative series of cases □✵
   b) potential for selection biases or not stated

3) Selection of Controls
   a) community controls □✵
   b) hospital controls
   c) no description

4) Definition of Controls
   a) no history of disease (endpoint) □✵
   b) no description of source

Comparability

1) Comparability of cases and controls on the basis of the design or analysis
   a) study controls for _______________ (Select the most important factor.) □✵
   b) study controls for any additional factor □✵ (These criteria could be modified to indicate specific control for a second important factor.)

Exposure

1) Ascertainment of exposure
   a) secure record (e.g. surgical records) □✵
   b) structured interview where blind to case/control status □✵
   c) interview not blinded to case/control status
   d) written self-report or medical record only
   e) no description

2) Same method of ascertainment for cases and controls
   a) yes □✵ b) no

3) Non-Response rate
   a) same rate for both groups ✵
   b) non respondents described
   c) rate different and no designation

Table S3. References included in the final analysis

Code	Reference information
1	Al-ani A, MacDonald D A, Ahmad M. Salivary sIgA and PRAP-1 Protein in Relation to Dental Caries: A Comparative Study[J]. Journal of Advanced Oral Research, 2020, 11(1): 71–76. https://doi.org/10.1177%2F2320206820913746
2	Haeri-Araghi H, Zarabadipour M, Safarzadeh-Khosroshahi S, et al. Evaluating the relationship between dental caries number and salivary level of IgA in adults[J]. Journal of Clinical and Experimental Dentistry, 2018, 10(1): e66-69. https://dx.doi.org/10.4317%2Fjced.54271
3	B. Jagadesh Babu, N. Venugopal Reddy, B. V. Thimma Reddy, et al. Comparitive evaluation of salivary IgA levels and dental caries in obese and non-obese children[J]. International Journal of Advanced Research，2017，5(1), 766-772. http://dx.doi.org/10.21474/IJAR01/2812
4	Bagherian A, Asadikaram G. Comparison of some salivary characteristics between children with and without early childhood caries[J]. Indian Journal of Dental Research, 2012, 23(5): 628-632. https://doi.org/10.4103/0970-9290.107380
5	Castro R J, Herrera R, Giacaman R A. Salivary protein characteristics from saliva of carious lesion-free and high caries adults[J]. Acta odontológica Latinoamericana, 2016, 29(2): 178-185. http://www.scielo.org.ar/pdf/aol/v29n2/v29n2a11.pdf
6	Chawda J G, Chaduvula N, Patel H R, et al. Salivary SIgA and dental caries activity[J]. Indian pediatrics, 2011, 48(9): 719-721. https://doi.org/10.1007/s13312-011-0113-y
7	Chopra M, Jadhav S, Venugopalan A, et al. Salivary immunoglobulin A in rheumatoid arthritis (RA) with focus on dental caries: a cross-sectional study[J]. Clinical rheumatology, 2012, 31(2): 247-250. https://doi.org/10.1007/s10067-011-1796-0
8	Doifode D, Damle S G. Comparison of salivary IgA levels in caries free and caries active children[J]. International Journal of Clinical Dental Science, 2011, 2(1):10-14. https://www.edentj.com/index.php/ijcds/article/view/196.
9	de Farias D G, Bezerra A C B. Salivary antibodies, amylase and protein from children with early childhood caries[J]. Clinical oral investigations, 2003, 7(3): 154-157. https://doi.org/10.1007/s00784-003-0222-7
10	Lo Giudice G, Nicita F, Militi A, et al. Correlation of s-IgA and IL-6 Salivary with Caries Disease and Oral Hygiene Parameters in Children[J]. Dentistry Journal, 2020, 8(1): 3. https://doi.org/10.3390/dj8010003
11	Golpasand Hagh L, Zakavi F, Ansarifar S, et al. Association of dental caries and salivary sIgA with tobacco smoking[J]. Australian Dental Journal, 2013, 58(2): 219-223. https://doi.org/10.1111/adj.12059
12	Hegde M, Devadiga D, Shetty C, et al. Correlation between dental caries and salivary immunoglobulin in adult Indian population: An in vivo study[J]. Journal of Restorative Dentistry, 2013, 1(1): 22-25. https://doi.org/10.4103/2321-4619.111229
13	Hocini H, Iscaki S, Bouvet J P, et al. Unexpectedly high levels of some presumably protective secretory immunoglobulin A antibodies to dental plaque bacteria in salivas of both caries-resistant and caries-

	susceptible subjects[J]. Infection and immunity, 1993, 61(9): 3597-3604. https://doi.org/10.1128/IAI.61.9.3597-3604.1993
14	Huang H H, Yu H, Zhang L, et al. Correlation between immunochemical level and patient with caries[J]. West China journal of stomatology, 2006, 24(1): 77-78. https://doi.org/10.3321/j.issn:1000-1182.2006.01.023 (in Chinese)
15	Kirtaniya B C, Chawla H S, Tiwari A, et al. Natural prevalence of antibody titres to GTF of S. mutans in saliva in high and low caries active children[J]. Journal of Indian Society of Pedodontics and Preventive Dentistry, 2009, 27(3): 135-138. https://doi.org/10.4103/0970-4388.57092
16	Kuriakose S, Sundaresan C, Mathai V, et al. A comparative study of salivary buffering capacity, flow rate, resting pH, and salivary Immunoglobulin A in children with rampant caries and caries-resistant children[J]. Journal of Indian Society of Pedodontics and Preventive Dentistry, 2013, 31(2): 69-73. https://doi.org/10.4103/0970-4388.115697
17	Letieri A S, Freitas-Fernandes L B, Valente A P C, et al. Longitudinal Evaluation of Salivary Iga-S in Children with Early Childhood Caries Before and After Restorative Treatment[J]. Journal of Clinical Pediatric Dentistry, 2019, 43(4): 239-243. https://doi.org/10.17796/1053-4625-43.4.3
18	Nawaz A, Batool H, Kashif M, et al. Immune profiling of saliva in patients with and without dental caries[J]. Bangladesh Journal of Medical Science, 2019, 18(3): 536-539. https://doi.org/10.3329/bjms.v18i3.41622
19	Omar O M, Khattab N M A, Rashed L A. Glucosyltransferase B, immunoglobulin a, and caries experience among a group of Egyptian preschool children[J]. Journal of Dentistry for Children, 2012, 79(2): 63-68. PMID: 22828760.
20	Pal S, Mitra M, Mishra J, et al. Correlation of total salivary secretory immunoglobulin A (SIgA) and mutans specific SIgA in children having different caries status[J]. Journal of Indian Society of Pedodontics and Preventive Dentistry, 2013, 31(4): 270-274. https://doi.org/10.4103/0970-4388.121831
21	Pandey S, Goel M, Nagpal R, et al. Evaluation of Total Salivary Secretory Immunoglobulin A and Mi/fans-specific SIgA among Children having Dissimilar Caries Status[J]. The journal of contemporary dental practice, 2018, 19(6): 651-655. https://doi.org/10.5005/jp-journals-10024-2314
22	Parisotto T M, King W F, Duque C, et al. Immunological and microbiologic changes during caries development in young children[J]. Caries research, 2011, 45(4): 377-385. https://doi.org/10.1159/000330230
23	Primasari A, Octiara E, Yanti N. Risk factor of secretory immunoglobulin A and salivary lysozyme level in children aged under 3 years to severe early childhood caries[C]. IOP Conference Series: Earth and Environmental Science. IOP Publishing, 2019, 305(1): 012001. https://iopscience.iop.org/article/10.1088/1755-1315/305/1/012001/meta
24	Priya P R G, Asokan S, Karthick K, et al. Effect of dental treatments on salivary immunoglobulin A of children with and without dental caries: a comparative study[J]. Indian Journal of Dental Research, 2013, 24(3): 394. https://doi.org/10.4103/0970-9290.118004

25	Ranadheer E, Nayak U A, Reddy N V, et al. The relationship between salivary IgA levels and dental caries in children[J]. Journal of Indian Society of Pedodontics and Preventive Dentistry, 2011, 29(2): 106-112. https://doi.org/10.4103/0970-4388.84681
26	Razi M A, Qamar S, Singhal A, et al. Role of natural salivary defenses in the maintenance of healthy oral microbiota in children and adolescents[J]. Journal of Family Medicine and Primary Care, 2020, 9(3): 1603-1607. https://doi.org/10.4103/jfmpc.jfmpc_1134_19
27	Shifa S, Muthu M S, Amarlal D, et al. Quantitative assessment of IgA levels in the unstimulated whole saliva of caries-free and caries-active children[J]. Journal of Indian Society of Pedodontics and Preventive Dentistry, 2008, 26(4): 158-161.https://doi.org/10.4103/0970-4388.44031
28	Soesilawati P, Notopuro H, Yuliati Y, et al. The role of salivary sIgA as protection for dental caries activity in Indonesian children[J]. Clinical, Cosmetic and Investigational Dentistry, 2019, 11: 291-295.https://doi.org/10.2147/CCIDE.S194865
29	Wu X, Liu S, Liu J. Investigation of relationship between secretory immunoglobulin A, salivary peroxidase and caries in children[J]. Journal of Modern Stomatology, 2004 (3): 199-201. https://doi.org/10.3969/j.issn.1003-7632.2004.03.003 (in Chinese)
30	Yassin H N. Comparison of immunoglobulin IgA level in the stimulated saliva of caries-free and caries-active children aged 7-10 years[J]. Journal of Baghdad College of Dentistry, 2016; 28(3):155-158. https://jcodental-uobaghdad-edu.org/index.php/jbcd/article/view/1443.
